# Understanding the hidden relations between pro- and anti-inflammatory cytokine genes in bovine oviduct epithelium using a multilayer response surface method

**DOI:** 10.1038/s41598-019-39081-w

**Published:** 2019-02-28

**Authors:** Rasoul Kowsar, Behrooz Keshtegar, Akio Miyamoto

**Affiliations:** 1 Department of Animal Science, College of Agriculture, Isfahan University of Technology, Isfahan, 84156–83111 Iran; 2 Graduate School of Animal and Food Hygiene, Obihiro University of Agriculture and Veterinary Medicine, Obihiro, Hokkaido, 080-8555 Japan; 3 Department of Civil Engineering, Faculty of Engineering, University of Zabol, P.B. 9861335-856, Zabol, Iran

## Abstract

An understanding gene-gene interaction helps users to design the next experiments efficiently and (if applicable) to make a better decision of drugs application based on the different biological conditions of the patients. This study aimed to identify changes in the hidden relationships between pro- and anti-inflammatory cytokine genes in the bovine oviduct epithelial cells (BOECs) under various experimental conditions using a multilayer response surface method. It was noted that under physiological conditions (BOECs with sperm or sex hormones, such as ovarian sex steroids and LH), the mRNA expressions of *IL*1*0*, *IL1B*, *TNFA*, *TLR4*, and *TNFA* were associated with *IL1B*, *TNFA*, *TLR4*, *IL4*, and *IL10*, respectively. Under pathophysiological + physiological conditions (BOECs with lipopolysaccharide + hormones, alpha-1-acid glycoprotein + hormones, zearalenone + hormones, or urea + hormones), the relationship among genes was changed. For example, the expression of *IL10* and *TNFA* was associated with (*IL1B*, *TNFA*, or *IL4*) and *TLR4* expression, respectively. Furthermore, under physiological conditions, the co-expression of *IL10* + *TNFA*, *TLR4* + *IL4*, *TNFA* + *IL4*, *TNFA* + *IL4*, or *IL10* + *IL1B* and under pathophysiological + physiological conditions, the co-expression of *IL10* + *IL4*, *IL4* + *IL10*, *TNFA* + *IL10*, *TNFA* + *TLR4*, or *IL10* + *IL1B* were associated with *IL1B*, *TNFA*, *TLR4*, *IL10*, or *IL4* expression, respectively. Collectively, the relationships between pro- and anti-inflammatory cytokine genes can be changed with respect to the presence/absence of toxins, sex hormones, sperm, and co-expression of other gene pairs in BOECs, suggesting that considerable cautions are needed in interpreting the results obtained from such narrowly focused *in vitro* studies.

## Introduction

The epithelial tissue of the female reproductive tract (FRT) reacts to different stimuli, such as pathogens, hormones, allogeneic sperm, semi-allogeneic embryo, and biochemical stressors; it secretes immune-related factors such as pro- and anti-inflammatory cytokines^1–4^ which are involved in the physiological or pathophysiological control of the oviduct function. For example, pro-inflammatory cytokines, such as interleukin (IL) 1B and tumor necrosis factor A (TNFA), play major roles in normal embryonic development and the transportation of gametes and embryo in the oviduct^5^. However, the over-expression of these pro-inflammatory cytokines could cause damages to the oviduct tissue^6,7^ and impairs early embryonic development^8,9^.

Various physiological or pathophysiological (abnormal) factors have been reported to change the balance between pro- and anti-inflammatory cytokine genes in the bovine oviduct epithelial cells (BOECs). We reported that urea, prostaglandin E2 (PGE2) and sperm cells would alter the expression pattern of cytokine genes from pro- to anti-inflammatory response in BOECs^2,4^. However, zearalenone disrupted the anti-inflammatory response of BOECs to sperm cells^10^. We have also reported that toxins, such as lipopolysaccharide (LPS) and zearalenone, induced a pro-inflammatory response in BOECs^10,11^. It is important to note that, these molecules may show similar interactions, the primary effects of each may vary depending on the hormonal changes during the ovarian cycle^1,10,12,13^. For example, Kowsar *et al*.^11^ and Fahey *et al*.^1^ reported that ovarian sex steroids inhibited LPS-induced expression of the pro-inflammatory mediators in BOECs and human uterine epithelial cells. These immune responses became more complicated when it was reported that a low dose of LPS (10 ng/mL) induced a pro-inflammatory response whereas high-dose LPS (100 ng/mL) favored an anti-inflammatory response in BOECs^11^. These findings reveal a complex regulation of the mRNA expression of pro- and anti-inflammatory cytokine genes based on the different experimental/biological conditions of the oviduct epithelium.

There is a well-known cross-regulation between pro- and anti-inflammatory cytokines (IL12 and IL1B versus IL4 and IL10, respectively)^14,15^ but this pattern sounds to be simple and cannot explain the exact relationship existing among the cytokine genes. A growing body of evidence shows that the pro- and anti-inflammatory responses are associated with each other^16,17^. For example, IL10 alone or with IL12 regulated the secretion of anti-inflammatory cytokines (such as IL4, IL5, and IL13) during infections^18^. In their study, van Kampen *et al*.^19^ reported that IL4 exhibited a pro-inflammatory role as in the case of colitis in mouse intestine and also co-expressed with TNFA. Furthermore, in addition to being a potent anti-inflammatory cytokine^18^, IL10 has been shown to have a pro-inflammatory function in an inflammatory environment *in vivo*^20^. Lauw *et al*.^21^ reported that IL10 enhanced the systemic concentration of inflammatory IFN-γ 1 h after endotoxin application. Hence, instead of being an anti-inflammatory cytokine, IL4 or IL10 may acquire the Janus-faced property, and thus showing a pro-inflammatory function based on the different conditions and environments, i.e., infections^19–22^. Therefore, understanding the gene-gene interactions under different biological or experimental conditions is of great importance and should be considered, especially in case of therapeutic treatment of infections if applicable^20,22^. Clearly, the study of the relationship among genes is experimentally expensive, time-consuming, and complicated^23^.

It is important to note that the pro- and anti-inflammatory cytokine pattern is mainly based on the data from the murine and human T-lymphocytes^24,25^ but little information exists on the exact relations among them in the bovine oviduct. Moreover, the available data mainly report that the specific stimulants simply favor skewness toward a pro-/anti-inflammatory status^3,4,9–11^. Computer-based systems are widely utilized by researchers for the medical prognoses, decision making, outcome prediction, and even for patient care^25–27^. Furthermore, bioinformatics approaches, such as clustering^28^, statistical techniques^29–31^ and nonlinear principal component analysis (PCA)^25,32,33^ have been utilized to interpret and understand the biological datasets, i.e., gene-gene interactions. However, most co-expression databases consider only the linearity (not nonlinearity) among genes and gene pairs^30,34^. For example, the Pearson and Spearman correlation coefficients cannot consider the nonlinearity among genes that show higher-order interactions^35,36^. This may hamper the precise evaluation of gene-gene relationships. Understanding such a gene-gene interaction or co-expression allows users to design the next experiments (hypothesis generation) more efficiently. This may also give a better understanding of the immunological responses in BOECs under various biological or experimental conditions.

We, therefore, hypothesized that the association between pro- and anti-inflammatory cytokine genes may change under different biological or experimental conditions in BOECs. Here, we first developed a highly nonlinear model and then investigated the direct and reciprocal relationships between gene expressions using 810 qRT-PCR data obtained from our classical experiments (16 independent BOECs cultures, four to five replications per each experiment).

## Results

### Comparative analysis of models

**Figure 1:**
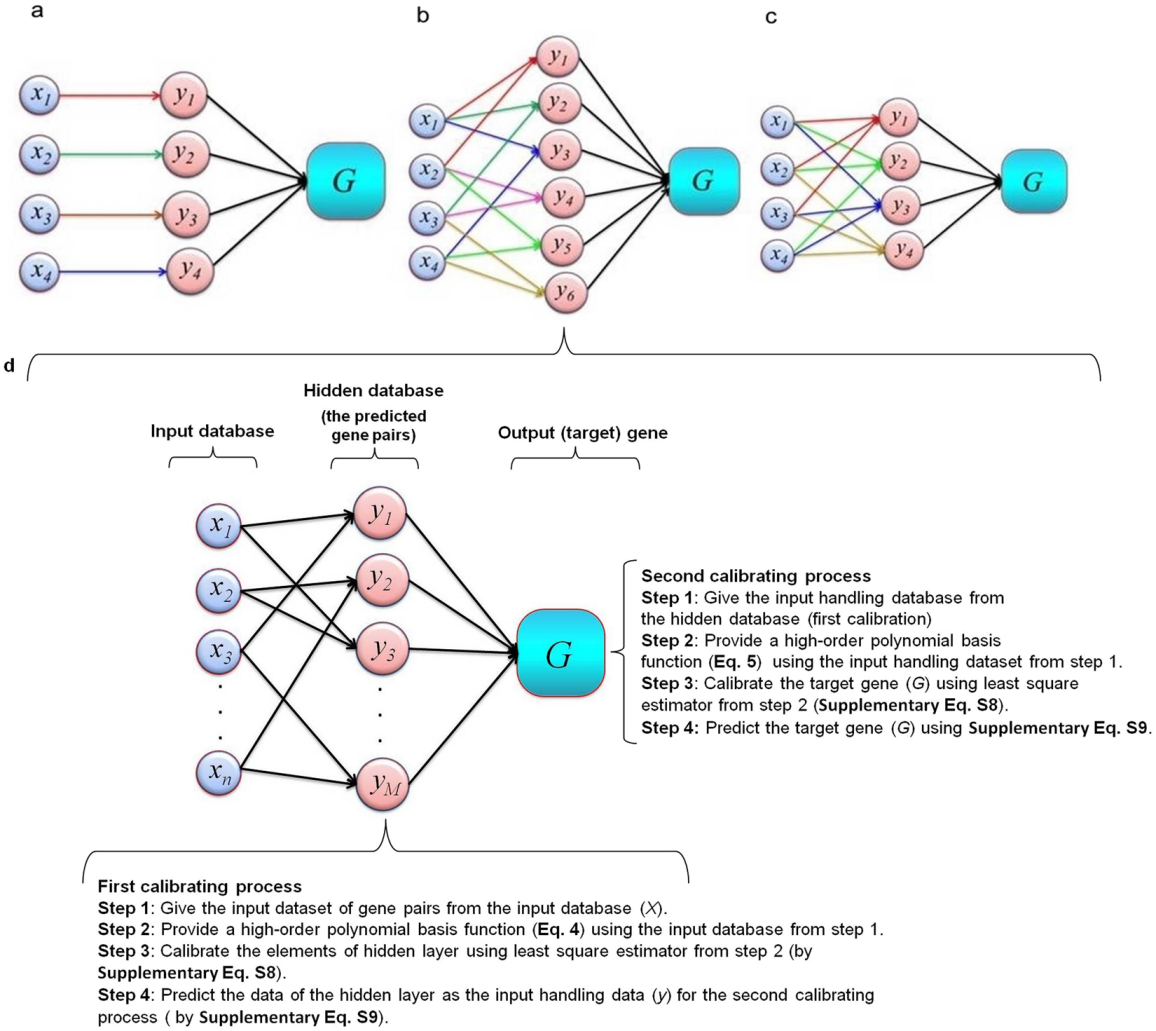
Schematic view of the different input gene sets for the hidden layer of MLRSM. (**a**) Scenario 1: the one-input dataset (*x*_1_*)* (for example, the gene expression data of *TNFA*) was used to calibrate the hidden predicted gene (*y*_*1*_). (**b**) Scenario 2: the two-input dataset (*x*_*1*_ and *x*_2_) (for example, the co-expression data of *TNFA* and *IL1B*) was used to calibrate the hidden predicted gene (*y*_*1*_). (**c**) Scenario 3: the three-input dataset (*x*_*1*_, *x*_*2*_, and *x*_3_) (for example, the gene expression data of *TNFA*, *IL*1*B*, and *IL4*) was applied for calibrating the elements of the hidden layer (*y*_1_). Then, *y*_*1*_ as a new input data was used for predicting the output/target gene (*G*) (for example, *TLR4*); and (**d**) this part provides a detail of the scenario 2. At the first calibration process, the hidden layer (*y*_1_ to *y*_*m*_) of the model was calibrated using the input genes (*x*_1_ *to x*_*n*_). Next, the output gene (*G*) was calibrated based on the calibrated dataset (the hidden layer) (*y*_1_ to *y*_*m*_). So, the second calibrating process was utilized to predict the output gene expression (*G*). Also, the data of the hidden layer of this model (gene pairs) was employed to construct Fig. 5a–e (the relationship between the co-expression of gene pairs and the output gene expression). The total elements in the hidden layer (*M*) were calculated based on the number of input genes (*NS*) and the total number of genes (*n*) as $M=\frac{n!}{(n-NS)!NS!}$ where! is the factorial operator and *NS* ≤ *n*. So, the elements of the hidden layer were computed *M* = 4 for scenario 1 based on *n* = 4 and *NS* = 1 (i.e. $M=\frac{n!}{(n-NS)!NS!}=\frac{4!}{(3)!\,1!}=4$); *M* = 6 for scenario 2 based on *n* = 4; and *NS* = 2 and *M* = 4 for scenario 3 based on *n* = 4 and *NS* = 3.

**Figure 2:**
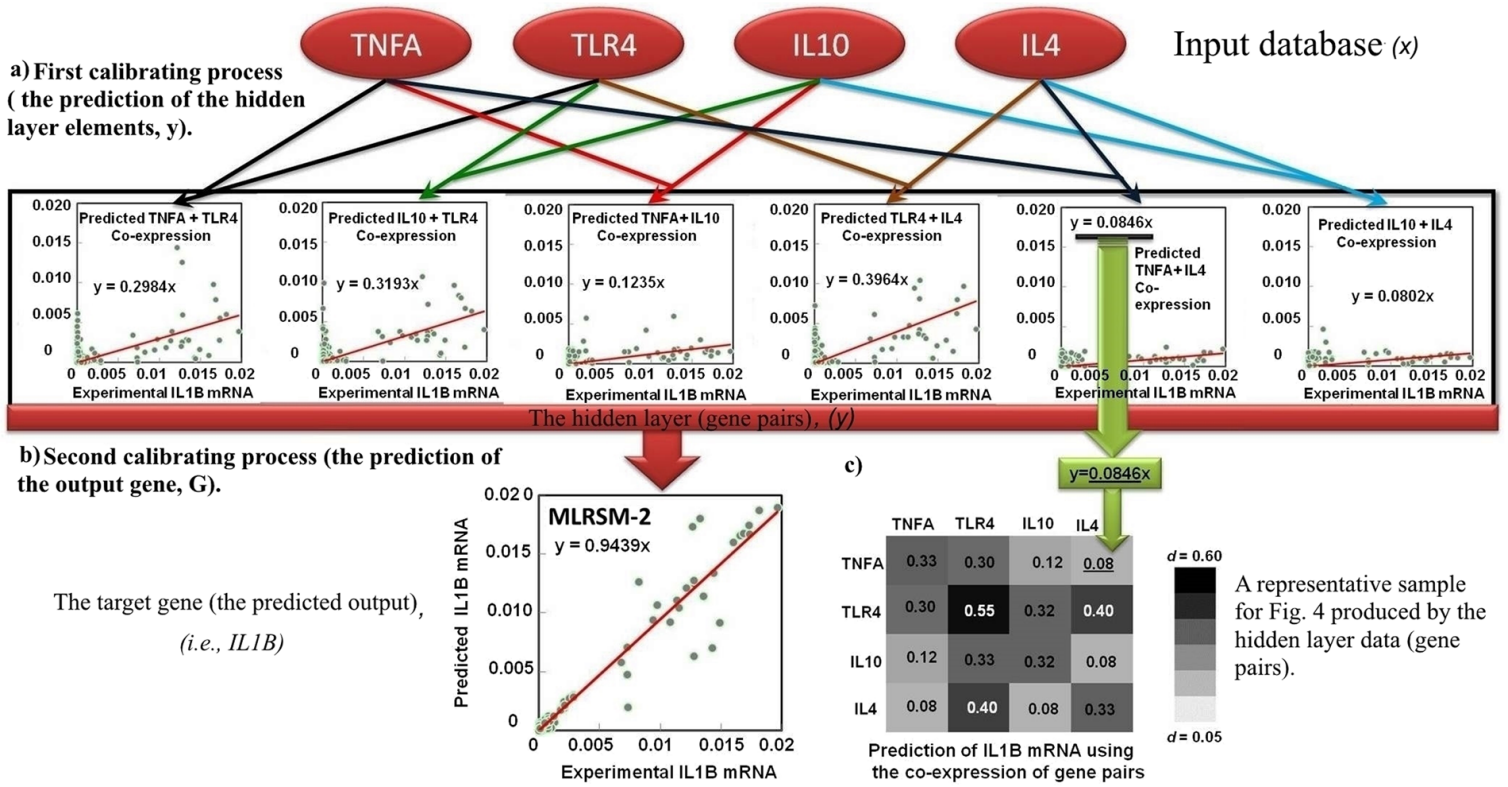
A representative structure of the MLRSM scenario 2 in predicting *IL1B* mRNA expression. (**a**) In the first calibrating process, a high-nonlinear polynomial function was employed to calibrate the hidden database (the predicted gene pairs) (*y*) by using the input dataset (*x*), i.e., *TNFA*, *TLR4*, *IL10*, and *IL4*, regardless to the experimental conditions. (**b**) The hidden database was shown as the predicted gene pairs, i.e., the co-expression of (*TNFA* + *TLR4*), (*IL10* + *TLR4*), (*TNFA* + *IL10*), (*TLR4* + *IL4*), (*TNFA* + *IL4*), or (*IL10* + *IL4*). Next, in the second calibrating process, the handling predicted data (the hidden layer, the predicted gene pairs) from the first calibrating process were employed for the regression analysis of the target (output) gene, *G*, (i.e., *IL1B*). (**c**) Due to the inclusion of the hidden layer to the first modeling approach; the model was able to consider the correlation between the input genes (*x*). Therefore, we utilized the data of the hidden layer (the predicted gene pairs) to construct the relations between gene pairs and output gene, i.e., *IL1B*, as seen in Fig. 5a–e).

In order to identify the best one, we developed three various models: (1) multiple-linear regression (MLR); (2) response surface method (RSM); and (3) multi-layer response surface method (MLRSM) using three different scenarios. We defined three scenarios for the hidden layer of the MLRSM based on the number of the input dataset as scenario 1 (the mRNA expression data of one gene was used as the input dataset for predicting the target gene, Fig. 1a), scenario 2 (the mRNA expression dataset of two genes (gene pairs) was used as the input dataset for predicting the target gene, Figs 1b,d and 2a), and scenario 3 (the mRNA expression dataset of three genes was used as the input dataset for predicting the target gene, Fig. 1c).

The comparative results are presented in Table 1. In order to identify the best model that fits the experimental data, the gene expression dataset of all genes was used (regardless of the experimental conditions). Using the mRNA expression data of all genes, the analysis showed that compared to the MLR, RSM, MLRSM (scenario 1) and MLRSM (scenario 3), the scenario 2 of the MLRSM model reduced (on average for all genes) the root-mean-square errors (*RMSE*) by 50.1%, 46.2%, 39.7%, and 21.8% and the mean bias error (*MBE*) by 67.3%, 72.7%, 57.7%, and 41.6%, respectively (Table 1). The scenario 2 of the MLRSM showed improved agreement index*, d*, compared to the MLR, RSM, MLRSM (scenario 1) and MLRSM (scenario 3) models by 151.2%, 81.7%, 30.7%, and 8.9%, respectively. Compared to the MLR, RSM, scenarios 1 and 3 of MLRSM, the scenario 2 showed improved goodness-of-fit (*EF*) between prediction and observation by 537.5%, 659.8%, 143.4%, and 23.2%, respectively.

**Table 1:** The comparative results of the different models: MLR, RSM, and MLRSM scenarios 1–3.

Gene	Statistics	MLR	RSM	MLRSM scenario 1	MLRSM scenario 2	MLRSM scenario 3
*IL1B*	*RMSE*	4.64E-3	4.27E-3	3.08E-3	**1.23E-3**	2.13E-3
	*MBE*	46.44	19.42	12.11	**0.84**	4.40
	*d*	0.42	0.59	0.85	**0.98**	0.95
	*EF*	0.11	0.25	0.61	**0.94**	0.81
*TNFA*	*RMSE*	1.11E-2	1.07E-2	9.53E-3	**6.84E-3**	7.46E-3
	*MBE*	2.70	2.61	2.63	**1.04**	1.05
	*d*	0.28	0.31	0.74	**0.88**	0.82
	*EF*	−0.05	0.03	0.23	0.34	**0.53**
*TLR4*	*RMSE*	3.41E-4	3.35E-4	2.96E-4	**2.52E-4**	2.73E-4
	*MBE*	0.95	0.53	0.28	**0.22**	0.19
	*d*	0.74	0.76	0.82	**0.89**	0.87
	*EF*	0.35	0.38	0.51	**0.65**	0.58
*IL10*	*RMSE*	9.12E-5	8.73E-5	7.83E-5	**3.69E-5**	4.19E-5
	*MBE*	533.95	517.29	17.00	**4.50**	6.95
	*d*	0.30	0.35	0.56	**0.95**	0.93
	*EF*	−0.13	−0.04	0.16	**0.81**	0.76
*IL4*	*RMSE*	3.73E-5	4.81E-5	3.06E-5	**1.76E-5**	2.87E-5
	*MBE*	1.91	−0.06	0.15	**0.09**	0.36
	*d*	0.34	0.41	0.65	**0.92**	0.71
	*EF*	−0.09	−0.81	0.27	**0.76**	0.35

Bold numbers are the best statistics obtained for each model as well as each gene. *RMSE* is the root-mean-square errors, *MBE* is the mean bias error, *EF* is the Nash-Sutcliffe efficiency, and *d* is Willmott’s index of agreement.

**Figure 3:**
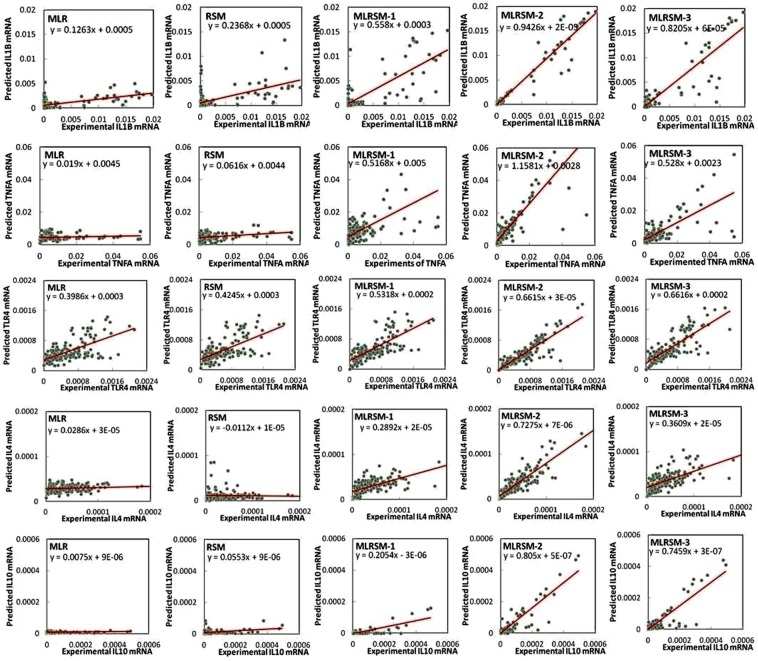
Scatterplots of the observed versus predicted gene expression data of different genes (*IL1B*, *TNFA*, *TLR4*, *IL10*, and *IL4*) using the MLR, RSM, and MLRSM (scenarios 1–3) models. We input entering-experimental values for making scatter plots. The entering data were obtained from 16 independent *in vitro* BOECs experiments with four to five replications. The scatterplots of the predicted and experimental data confirmed a strong nonlinear relationship among the candidate genes. Considering the scatterplots, scenario 2 showed a better prediction and evaluated a proper coefficient for the input data points.

Figure 3 shows the scatterplots of the predicted and experimental mRNA expression data obtained using MLR, RSM and MLRSM (scenarios 1 to 3) models. The scatterplots indicated that all scenarios of the MLRSM showed improved slope lines while scenario 2 of MLRSM showed the better prediction of the mRNA expression of all genes. The correlations (*a*) of the MLR model ranged from 0.007 to 0.399 while that of RSM model was from 0.011 to 0.425. The correlations (*a*) of the MLRSM model ranged from 0.205 to 0.558 for scenario 1, from 0.661 to 1.158 for scenario 2, and from 0.360 to 0.821 for scenario 3. Thus, scenario 2 showed a better prediction and a proper coefficient for the input data points evaluated and was used to investigate gene-gene interaction.

### Nonlinear-based principal component analysis (nonlinear PCA) to detect the prediction accuracy and main mRNA expression pattern

**Figure 4:**
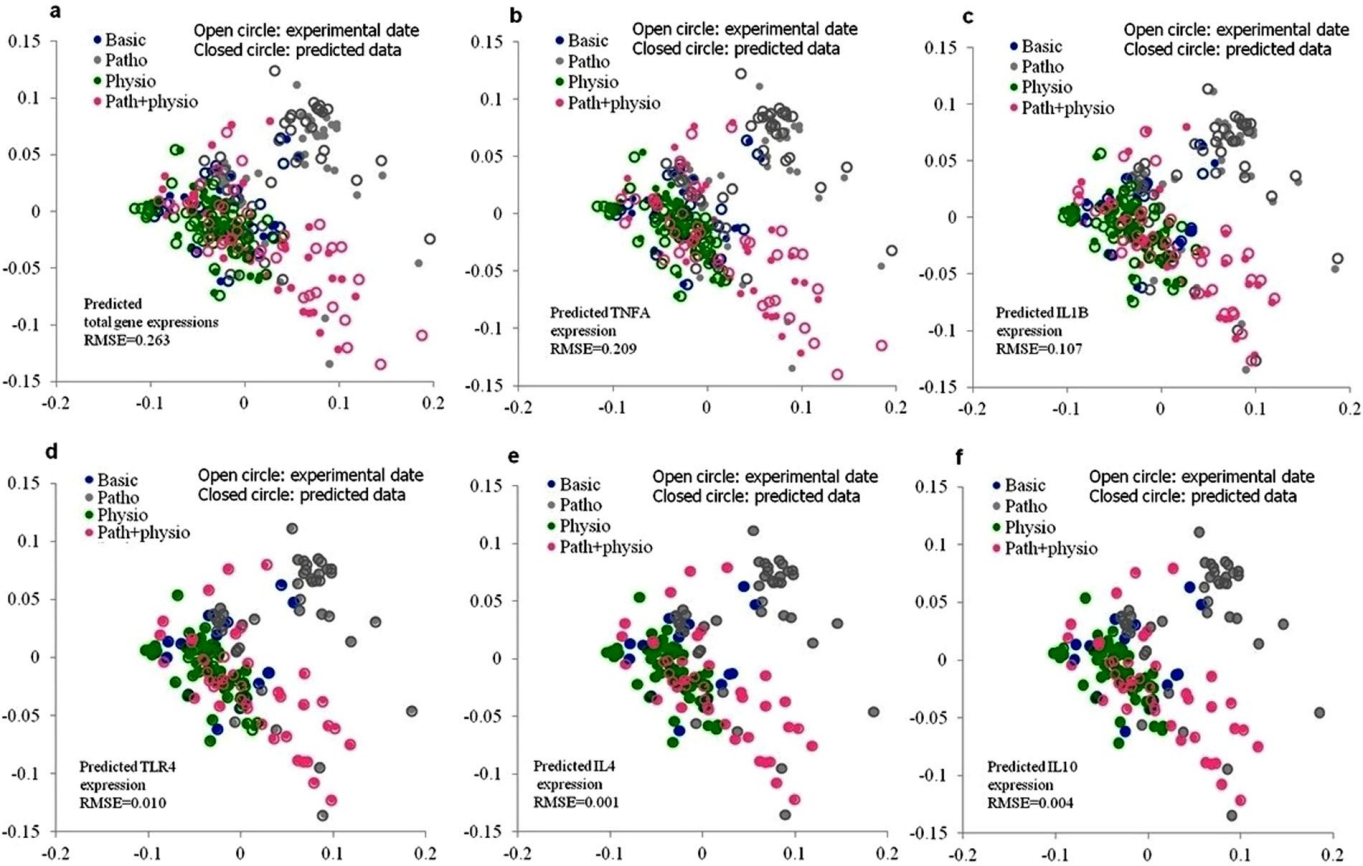
Nonlinear-based principal component analysis, nonlinear PCA, was applied to the experimental and MLRSM-2-predicted gene expression data to evaluate the performance of the MLRSM scenario 2 in predicting the gene expression of candidate genes. The experimental and MLRSM-2-predicted gene expression data are shown by closed circles and open circles, respectively. The nonlinear PCA revealed a good association between each predicted data point and corresponding actual data point of *IL10*, *IL4*, *TLR4*, *IL1B*, and *TNFA* (*RMSE* values were low and ranged from 0.004 to 0.209 for these genes). Also, the nonlinear PCA exhibited that, the mRNA expression of candidate genes under experimental conditions (physiological, pathophysiological, or pathophysiological + physiological conditions) was similarly predicted by the predictor (the MLRSM scenario 2). This suggested the similarity of gene expression patterns within samples obtained from the same experimental conditions, implying a proper preparation and choice of BOECs samples. Basic: un-stimulated BOECs culture; Patho: pathophysiological condition; Physio: physiological condition; Path + physio: pathophysiological + physiological condition. *RMSE* is the root-mean-square errors.

The nonlinear PCA^25^ was employed to examine the predictor performance (i.e., scenario 2) and detect how close the predicted data were to the experimental data. The scenario 2-predicted data of all genes were projected into the nonlinear-based PCA and compared against the experimental data (Fig. 4a). It was found that the nonlinear-based PCA showed a high variation and root-mean-square errors (*RMSE* = 0.263) both in the experimental (***O****i*) and MLRSM (scenario 2)-predicted gene expression data (***P****i*) as $RMSE=\sqrt{\frac{1}{N}\sum _{i=1}^{N}\Vert {{\boldsymbol{O}}}_{i}-{{\boldsymbol{P}}}_{i}\Vert {]}^{2}}$.

Next, the scenario 2-predicted data of each gene was projected into the nonlinear PCA and compared against the experimental data of all genes (Fig. 4b–f). The result revealed that scenario 2 was not good at predicting the mRNA expression of *TNFA* (*RMSE* = 0.209) and *IL*1*B* (*RMSE* = 0.107) compared to other candidate genes. While on the other hand, the nonlinear-based PCA detected the superior performance of the predictor (scenario 2) in predicting the mRNA expression of *IL4* (*RMSE* = 0.001), *IL*1*0* (*RMSE* = 0.004), and *TLR4* (*RMSE* = 0.010); and also detected the likeness in predicting the mRNA expression of all genes, under physiological, pathophysiological, or pathophysiological + physiological conditions.

### The relationship between the mRNA expression of *IL1B* and other genes under various experimental conditions of BOECs

As shown in Table 2, we identified a strong correlation (*P* < 0.05) between *IL1B* expression and *TLR4* (*d* = 0.36) or *TNFA* expression (*d* = 0.27) under basic conditions. Data revealed that the expression data of *TLR4* (*d* = 0.60) or *TNFA* (*d* = 0.50) could provide the best prediction for *IL1B* expression under pathophysiological conditions (*P* < 0.05). Under physiological conditions, the model showed a positive relationship (*P* < 0.05) between mRNA expression of *IL1B* with *IL10* (*d* = 0.17), *TNFA* (*d* = 0.15), and *IL4* (*d* = 0.11) expression. The expression of *IL1B* was positively associated with *IL10* (*d* = 0.23) and *TNFA* (*d* = 0.17) expression but negatively associated with *IL4* expression (*d* = 0.17) under pathophysiological + physiological conditions (*P* < 0.05).

**Table 2:** The relationship between different genes under various experimental conditions of BOECs predicted by MLRSM (scenario 2).

Experimental condition	*IL1B*				*TNFA*				*TLR4*			
	Gene	*d*	*AIC*	P-value	Gene	*d*	*AIC*	P-value	Genes	*d*	*AIC*	P-value
Basic	TNFA	**0.27**	11.9	**0.04**	IL1B	**0.53**	16.5	**6E-3**	IL1B	**0.55**	13.9	**0.02**
	TLR4*	**0.36**	14.3	**0.01**	TLR4	0.30	6.2	0.29	TNFA*	**0.60**	16.6	**5E-3**
	IL10	0.17 (−)	8.7	0.15	IL10*	**0.51** (−)	15.8	**0.01**	IL10	0.21 (−)	7.0	0.22
	IL4*	0.21 (−)	8.9	0.11	IL4*	**0.50 (−)**	13.4	**0.02**	IL4*	0.23 (−)	10.7	0.06
Physio.	TNFA*	**0.15**	11.9	**0.04**	IL1B	**0.49**	13.5	**0.02**	IL1B	**0.50**	12.2	**0.03**
	TLR4	0.04	6.3	0.28	TLR4*	0.17	5.0	0.42	TNFA*	**0.55**	14.4	**0.01**
	IL10*	**0.17**	10.9	**0.01**	IL10	**0.46**	12.1	**0.03**	IL10	0.28	8.6	0.12
	IL4	**0.11**	14.6	**0.05**	IL4*	0.27	8.2	0.14	IL4*	**0.55**	13.5	**0.02**
Patho.	TNFA	**0.50**	11.5	**0.04**	IL1B	**0.23**	11.5	**0.04**	IL1B*	**0.82**	31.3	**8E-5**
	TLR4*	**0.60**	21.1	**8E-4**	TLR4*	0.18 (−)	8.1	0.15	TNFA*	0.47	8.7	0.12
	IL10	0.25	5.1	0.40	IL10*	**0.23 (−)**	10.5	**0.04**	IL10	0.43 (−)	9.7	0.08
	IL4*	0.29 (−)	4.8	0.43	IL4	0.21 (−)	10.2	0.07	IL4	0.47 (−)	9.2	0.10
Patho + physio	TNFA	**0.17**	23.2	**2E-**3	IL1B	**0.44**	11.9	**0.04**	IL1B	**0.30**	11.3	0.05
	TLR4	0.14 (−)	9.9	0.08	TLR4	0.39	6.6	0.25	TNFA*	**0.47**	23.2	**3E-4**
	IL10*	**0.23**	22.8	**4E-4**	IL10*	**0.61**	28.6	**3E-5**	IL10*	0.28 (−)	7.6	0.18
	IL4*	**0.17** (−)	25.7	**2E-3**	IL4*	0.40 (−)	10.7	0.06	IL4	0.19 (−)	5.1	0.40
**Experimental condition**	***IL4***				***IL10***							
	**Gene**	***d***	***AIC***	**P-value**	**Gene**	***d***	***AIC***	**P-value**				
Basic	IL1B*	**0.57 (−)**	15.5	8E-3	IL1B	0.08 (−)	9.7	0.08				
	TNFA	0.33	6.9	0.23	TNFA*	**0.61** (−)	15.1	**0.01**				
	TLR4	**0.37 (−)**	**11.0**	**0.05**	TLR4*	**0.34 (−)**	11.3	**0.05**				
	IL10*	**0.55 (−)**	12.6	**0.03**	IL4	0.04 (−)	9.1	0.11				
Physio	IL1B*	**0.51**	**13.0**	**0.02**	IL1B	**0.41**	12.2	**0.03**				
	TNFA	**0.47**	11.3	**0.05**	TNFA*	**0.41**	11.6	**0.04**				
	TLR4	**0.53**	15.4	**9E-3**	TLR4	0.35	6.6	0.25				
	IL10*	0.41	10.4	0.07	IL4*	0.26	3.5	0.62				
Patho	IL1B*	**0.40 (−)**	12.1	**0.03**	IL1B*	**0.15**	12.9	**0.02**				
	TNFA	**0.40 (−)**	**11.2**	**0.05**	TNFA*	**0.17 (−)**	13.8	**0.02**				
	TLR4	0.13 **(−)**	7.9	0.16	TLR4	0.05	10.9	0.55				
	IL10*	**0.40 (−)**	10.2	0.05	IL4	1E-3 **(−)**	9.2	0.60				
Patho + physio	IL1B*	**0.40 (−)**	36.0	**9E-07**	IL1B	**0.37**	13.5	**0.02**				
	TNFA	**0.35 (−)**	18.5	**2E-3**	TNFA*	**0.43**	31.0	**1E-5**				
	TLR4	0.32 (−)	10.6	0.06	TLR4*	0.31 (−)	4.3	0.51				
	IL10*	**0.51 (−)**	**37.9**	**4E-07**	IL4	0.30 (−)	3.8	0.58				

The asterisk sign (*) implies that under each experimental condition (basic, physiological, etc.), the mRNA expression of these gene pairs is significantly associated with the target gene. The minus sign (−) means that the reciprocal relationship between this gene and the target gene is negative otherwise the relationship is positive (the Pearson correlation analyses were conducted using Pearson’s rank correlation analysis method to evaluate the negative or positive relationship among the candidate genes). Basic: un-stimulated BOECs culture; Patho: pathophysiological condition; Physio: physiological condition; Patho + physio: pathophysiological + physiological condition. *AIC* (*ΔAIC*) is the Akaike Information Criteria; *d* is Willmott’s index of agreement applied to make a cross-comparison between predicting models and observed data points in the range from 0 (no correlation) to 1 (perfect correlation).

### The relationship between mRNA expression of *TNFA* and other genes under various experimental conditions of BOECs

Under basic conditions, the mRNA expression of *IL1B* (positively) (*d* = 0.53), *IL10* (negatively) (*d* = 0.51), and *IL4* (negatively) (*d* = 0.50) provided a significant prediction for *TNFA* expression (Table 2). Under physiological conditions, *IL1B* (*d* = 0.49) and *IL10* (*d* = 0.46) exhibited the highest positive association (*P* < 0.05) with the *TNFA* mRNA expression. Under pathophysiological conditions, the mRNA expression of *IL1B* (*d* = 0.23, positively) and that of *IL10* (*d* = 0.23, negatively) provided a good prediction for *TNFA* expression (*P* < 0.05); while under pathophysiological + physiological conditions, the expression data of *IL10* (*d* = 0.61) or *IL1B* (*d* = 0.44) was positively correlated with *TNFA* expression.

### The relationship between mRNA expression of *TLR4* and other genes under various experimental conditions of BOECs

As shown in Table 2, the mRNA expression of *TNFA* was positively associated (*P* < 0.05) with *TLR4* expression under basic (*d* = 0.60) and pathophysiological + physiological (*d* = 0.47) conditions. Also, it was observed that the mRNA expression of *IL1B* provided a significant positive prediction for *TLR4* expression under basic (*d* = 0.55) and pathophysiological + physiological (*d* = 0.30) conditions (*P* < 0.05).

Under physiological conditions, the mRNA expression of *TLR4* was positively correlated (*P* < 0.05) with *TNFA* (*d* = 0.55), *IL4* (*d* = 0.55), and *IL1B* expression (*d* = 0.50). Under pathophysiological conditions, the expression data of *IL1B* provided a good prediction (*d* = 0.82) for *TLR4* expression (*P* < 0.05).

### The relationship between mRNA expression of *IL4* and other genes under various experimental conditions of BOECs

A negative correlation was detected (*P* < 0.05) between *IL4* mRNA expression and the expression data of *IL1B* (*d* = 0.57), *IL10* (*d* = 0.55), and *TLR4* (*d* = 0.37) under basic conditions (Table 2). Under physiological conditions, the mRNA expression data of *IL4* was positively associated (*P* < 0.05) with *TLR4* (*d* = 0.53), *IL1B* (*d* = 0.51), and *TNFA* (*d* = 0.47). The expression data of *IL1B*, *IL10*, and *TNFA* (*d* = 0.40) was negatively correlated with the mRNA expression of *IL4* under pathophysiological conditions (*P* < 0.05); while under pathophysiological + physiological conditions, the mRNA expression data of *IL4* exhibited a negative correlation (*P* < 0.05) with *IL10* (*d* = 0.51), *IL1B* (*d* = 0.40), and *TNFA* (*d* = 0.35).

### The relationship between mRNA expression of *IL10* and other genes under various experimental conditions of BOECs

The results showed that *TNFA* (*d* = 0.61) and *TLR4* (*d* = 0.34) were negatively associated with *IL10* expression under basic conditions (*P* < 0.05). The expression data of *IL10* was positively correlated (*P* < 0.05) with *IL1B* (*d* = 0.41) and *TNFA* (*d* = 0.41) expression under physiological conditions. Also, under pathophysiological conditions, we identified a positive correlation (*d* = 0.15) between *IL10* and *IL1B* (*P* < 0.05), but under this condition, *IL10* was negatively correlated (*P* < 0.05) with *TNFA* expression (*d* = 0.17). The results showed a positive correlation between *IL10* expression and *IL1B* (*d* = 0.37) or *TNFA* (*d* = 0.43) under pathophysiological + physiological conditions (*P* < 0.05) (Table 2).

The non-significant relationship between genes estimated by Akaike Information Criterion (*AIC*) was provided in the Supplementary text.

### The relationship between the mRNA expression of each gene and the co-expression of other gene pairs

**Figure 5:**
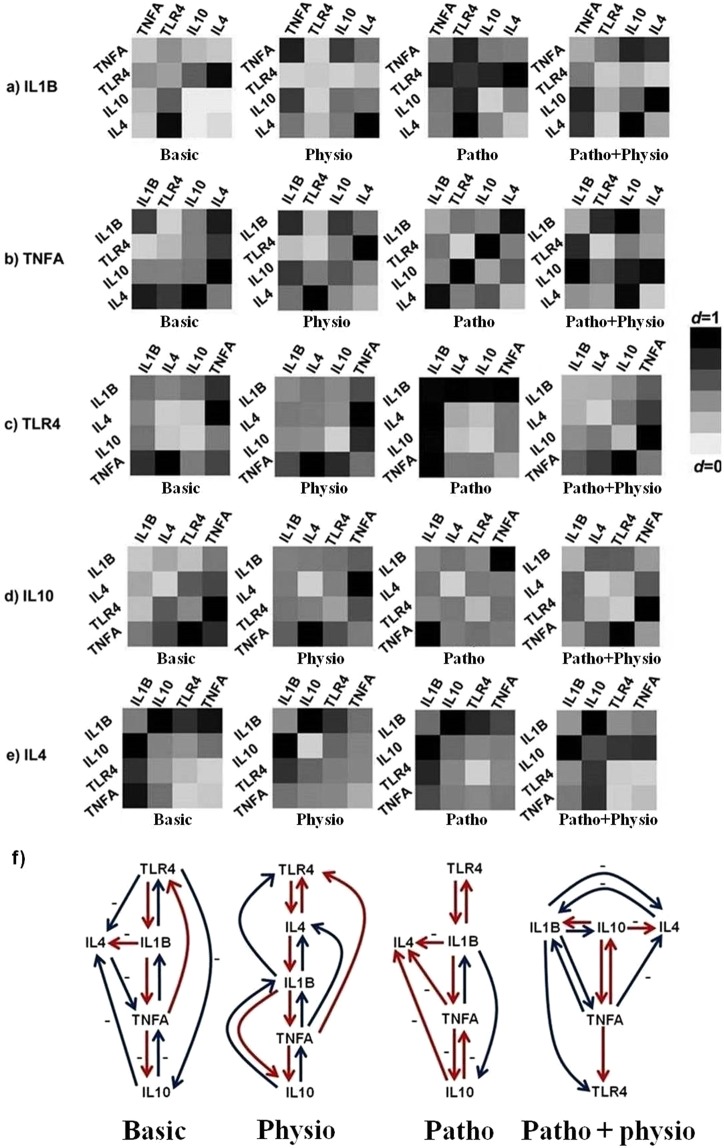
(**a**–**e**) The relationship between the co-expression of gene pairs and the output gene. The scenario 2 of the MLRSM (at which two-input datasets were used to calibrate the hidden genes) was utilized to predict the expression of each gene using the mRNA expression of gene pairs while considering the nonlinearity among them. The data of the hidden layer (gene pairs) of this model was employed to construct the relationship between the co-expression of gene pairs and the output gene as shown in Fig. 2c). (**f**) The schematic view of the relationships among the candidate genes under different experimental conditions predicted by the MLRSM model, scenario 2. This figure shows which gene (mRNA expression data) provides a good prediction for other genes at each experimental condition. The minus sign (−) means a negative correlation between this gene and the target gene otherwise the relationship is a positive one (the Pearson correlation analysis was conducted to test the negative or positive direction of the relationships among the genes). Both red and blue lines indicate a significant relationship calculated by *AIC*. But, compared with blue lines, the red lines show a stronger relationship. Basic: un-stimulated BOECs culture; Patho: pathophysiological conditions; Physio: physiological conditions; Patho + physio: pathophysiological + physiological conditions. *d* is Willmott’s index of agreement applied to make a cross-comparison between predicting models and observed data points in the range from 0 (no correlation, the brightest) to 1 (perfect correlation, the darkest).

The results showed that the best prediction of *IL1B* expression is obtained from the co-expression of *TLR4* and *IL4* under basic and pathophysiological conditions. The co-expression of *IL10* and *TNFA* (under physiological conditions) and the co-expression of *IL10* and *IL4* (under pathophysiological + physiological conditions) provided the best prediction for *IL1B* expression (Fig. 5a and Table 2).

Under basic conditions, the results showed a correlation between *TNFA* and the co-expression of *IL10* and *IL4*. Data revealed that the *TNFA* expression was associated with the co-expression of *TLR4* and *IL4* under physiological conditions. The co-expression of *TLR4* and *IL10* (under pathophysiological conditions) and the co-expression of *IL10* and *IL4* (under pathophysiological + physiological conditions) were the best predictors for *TNFA* expression (Fig. 5b and Table 2).

We found that the expression of *TLR4* was associated with the co-expression of *TNFA* and *IL4* under both basic and physiological conditions. But, under pathophysiological conditions, the co-expression of *IL1B* and *TNFA* predicted the best expression of *TLR4*. The results showed that there was a strong association between *TLR4* expression and the co-expression of *TNFA* and *IL10* under pathophysiological + physiological conditions (Fig. 5c and Table 2).

Under basic and pathophysiological + physiological conditions, we detected a strong association between *IL10* expression and the co-expression of *TNFA* and *TLR4*. The co-expression of *TNFA* and *IL4* (under physiological conditions) and the co-expression of *TNFA* and *IL1B* (under pathophysiological conditions) provided the best prediction for *IL10* expression (Fig. 5d and Table 2). The results showed that the expression of *IL4* was associated with the co-expression of *IL10* and *IL1B* under all experimental conditions (Fig. 5e and Table 2).

## Discussion

In the present study, we sought to identify the relationship between the mRNA expression of some pro- and anti-inflammatory cytokine genes and their co-expression by other gene pairs under various experimental conditions of BOECs *via* developing a highly nonlinear model, MLRSM scenario 2. (Please see detailed model Discussion in the Supplementary text).

In the present study, we discovered that the mRNA expression of *TLR4* provided the best prediction (positively correlated) for *IL1B* under pathophysiological conditions (Fig. 5f and Table 2). Interestingly, either in the physiological conditions (in the presence of sperm cells and the pre-ovulatory levels of sex hormones around the ovulation time) or in the pathophysiological + physiological conditions (in the presence of LPS/toxins and the pre-ovulatory levels of ovarian sex steroids, LH, or sperm cells), the mRNA expression of *IL1B* was accurately predicted by *IL10* mRNA expression, but not *TLR4* (Fig. 5f and Table 2). Physiologically, the positive correlation of *IL10* and *IL1B* implies that they may play a certain role in early reproductive events in the oviduct. For example, Huang *et al*.^37^ reported that the mRNA expression of *IL1B* was reduced in human oviduct during an ectopic pregnancy. Also, it has been shown that IL10 regulates fetal extracellular matrix^38^, and competent oocytes had a higher level of IL10 in their follicular fluids^39^ than their counterparts that failed to reach the blastocyst stage. Pathophysiologically, the positive correlation between *IL10* and *IL1B* expression under pathophysiological + physiological conditions suggested that IL10 as part of an immune-regulatory system may reduce the detrimental effects of pro-inflammatory IL1B on the oviduct tissue^6,7^ or embryo^8,9^.

Using the co-expression data of other gene pairs, it was discovered that the co-expression of *TLR4* and *IL4* provided a good prediction for *IL1B* under pathophysiological conditions (assaulting with infections or toxins); whereas the co-expression of *IL10* and *IL4* was associated with the *IL1B* expression under pathophysiological + physiological conditions. Two possibilities can be considered with respect to these associations. On the one hand, it reveals that *IL4* and *IL10* (the anti-inflammatory cytokines)^40^ could inhibit *TLR4* transcription, reduce LPS responsiveness, and consequently decrease the expression of pro-inflammatory cytokines, i.e., *IL1B*. This might imply a role for ovarian sex steroid hormones, LH, and sperm to induce a compensatory function by favoring *IL4* and *IL10* co-expression, and thus reducing the expression of pro-inflammatory cytokines, i.e., *IL1B*, in BOECs during abnormal conditions (i.e., the presence of LPS or toxins)^12,41^. Similarly, the experimental data showed that a high dose of LPS increased the expression of *IL4* and *IL10* in BOECs^11^, implying that LPS can weaken the host immune system, leading to a persistent infection^42,43^.

On the other hand, under specific conditions, IL4 and IL10 have been shown to have a pro-inflammatory property^19–22^. For example, IL10 in interaction with IFN-γ exhibited a pro-inflammatory effect^20^. Also, IL4 has been shown to induce the expression of pro-inflammatory IL6 in human endothelial cells^22^. Hence, in the presence of toxins (LPS, zearalenone, and urea), ovarian sex steroids and LH in BOECs culture, the exact outcome of the association between *IL4*, *IL10*, and *IL1B* needs further investigations.

Moreover, the co-expression of *IL10* and *TNFA* was highly correlated with *IL1B* expression under physiological conditions, suggesting a physiological role for these cytokines in the presence of the pre-ovulatory levels of ovarian sex steroids and LH, as well as sperm cells, around the ovulation time. For example, it has been suggested that TNFA is involved in the oviduct contraction and embryo transportation^5^. Moreover, Sinzato *et al*.^44^ reported that a low plasma level of IL10 was associated with impaired early embryonic development in rats. It was also reported in a study that the mRNA expression of *IL1B* was decreased in human oviduct during ectopic pregnancies^37^. Chaubey *et al*.^45^ reported that the cleavage and blastocyst rates were increased in buffalo oocytes upon supplementation of the maturation medium with IL1B. Therefore, the present data suggest that the pre-ovulatory levels of ovarian sex steroid hormones and LH, as well as the presence of sperm cells, may provide an optimal cytokine pattern (both pro- and anti-inflammatory cytokines) to support early reproductive processes.

Interestingly, the expression of *IL1B* provided the best prediction (positive correlation) for *TNFA* expression under pathophysiological conditions. It seems that *TLR4* expression, first, regulated *IL1B* expression and then indirectly controlled *TNFA* expression through *IL1B* expression. Guijarro-Muñoz *et al*.^46^ reported that the treatment of the human brain vascular pericytes with LPS induced the expression of *IL1B* (compared to *TNFA*) to a significant extent. The data, therefore, showed a hidden relationship between *TLR4*, *ILB*, and *TNFA* under pathophysiological conditions of BOECs; whereas classical studies only reported that LPS stimulation could generally induce the gene expression of *IL1B*, *TLR4* and *TNFA*^3,4,9–11,47^. For example, a low dose of LPS (10 ng/mL) or zearalenone induced the mRNA expression of *IL1B*, *TLR4* and *TNFA* in BOECs^10,11^ and in the rat synovial fibroblasts^47^. Understanding these hidden relationships between genes may help us to effectively make/reach a decision, as well as to design future experiments.

The present study not only showed the hidden relationships among genes in the same experimental conditions but also exhibited the hidden changes in gene-gene relations under different experimental conditions. For example, under pathophysiological + physiological conditions the relationship between *IL10* and *TNFA* was positively correlated; while, under pathophysiological conditions, the association was negatively correlated (Fig. 5f and Table 2). Zhai *et al*.^48^ reported similar results in a study where both positive and negative relationships existed between *TNFA* and *IL10* expression. The present data implied that physiological stimuli, such as ovarian sex steroids (alone or in the presence of LPS or other toxins) and sperm cells, altered the relationship between *IL10* and *TNFA* from a negative to positive association. Moreover, the positive relation of *IL10* with *TNFA* under physiological conditions implied that these cytokines play a role in the normal function of the oviduct in regulating gametes and embryo transportation as reported by others^5^.

Moreover, the positive association between *IL10* and *TNFA* (under pathophysiological + physiological conditions) implied a potential counter-regulatory loop in inhibiting an uncontrolled pro-inflammatory response and reducing tissue damage^6,7^ which could be caused by pathogens, LPS or toxins^1,12,41,43^. Armstrong *et al*.^49^ reported that IL10 reduced the mRNA expression of *TNFA* by LPS-treated macrophages. So, increased expression of *IL10* can reduce the pro-inflammatory response and meanwhile may impair the clearance of pathogens in case of acute infections^50^, predisposing the oviduct to a persistent infection and salpingitis^42,43,51^. On the other hand, the negative association between *IL10* and *TNFA* during pathophysiological conditions (considering that under this experimental condition, there were no ovarian sex steroids and LH in the culture medium) indicated that with an increase in pro-inflammatory *TNFA* expression, the expression of anti-inflammatory *IL10* decreased. This may result in a detrimental pro-inflammatory response in BOECs. The data, therefore, revealed an important role of the ovarian sex steroid hormones and LH as a part of BOECs homeostasis in controlling the over-expression of detrimental pro-inflammatory cytokines during infections.

Furthermore, the results showed that the co-expression of *IL10* and *IL4* accurately predicted the *TNFA* expression under pathophysiological + physiological conditions, as seen in (Fig. 5b and Table 2). This may again buttress that the expression of anti-inflammatory cytokines, such as *IL10* and *IL4*, is necessary to prevent the overwhelming pro-inflammatory response against host cells. However, the potential involvement of the *IL10* or *IL4* expression in the pro-inflammatory response of BOECs should be investigated^19–22^.

The expression of *TLR4* was associated with the mRNA expression of *TNFA* (under physiological or pathophysiological + physiological conditions), *IL4* (under physiological conditions), and *IL1B* (under pathophysiological conditions). Rycke *et al*.^52^ similarly reported that the blockade of *TNFA* resulted in a strong suppression of *TLR4*. Moreover, using the co-expression data of gene pairs, the data showed that the co-expression of *IL1B* and *TNFA* (under pathophysiological conditions), the co-expression of *TNFA* and *IL4* (under physiological conditions), and the co-expression of *TNFA* and *IL10* (under pathophysiological + physiological conditions) was strongly associated with *TLR4* expression. These findings suggest that the expression of *TNFA* was mainly associated with *TLR4* expression in BOECs under various experimental conditions. However, in the presence of the pre-ovulatory levels of ovarian sex steroid hormones, LH or sperm cells, the anti-inflammatory cytokines (*IL10* and *IL4*) affected the prediction of *TLR4* expression. Interestingly, the expression of *TLR4* was positively associated with *IL4* expression under physiological conditions (Table 2). We also discovered that *TLR4* mRNA was up-regulated in the pre-ovulatory oviducts (*in vivo*, unpublished data). It has been shown in similar studies that the physiological levels of estradiol increased *Tlr4* mRNA in mice macrophage^53^. Considering that IL4 can inhibit *TLR4* transcription *via* acting on the STAT6 binding site within the TLR4 gene^54^, it can, therefore, be suggested that *IL4* as a potent anti-inflammatory mediator may act in a counter-regulatory manner to regulate the over-expression of *TLR4* caused by ovarian sex steroid hormones, especially estradiol^53^, during the pre-ovulatory phase^11^.

Based on the negative correlation observed between anti-inflammatory cytokines (*IL10* and *IL4*) and *TLR4* expression under pathophysiological + physiological conditions (Table 2), it can be suggested that another role exists for the ovarian sex steroids, LH and sperm cells in inducing *IL10* and *IL4* expression, regulating *TLR4*, and thus reducing the overwhelming pro-inflammatory response^40^. This result confirmed the experimental data that high dose of LPS (100 ng/mL) or a high level of urea (40 mg/dL) either in the presence or absence of ovarian sex steroids and LH would induce the gene expression of *IL4* and *IL10* but reduce *IL1B*, *TNFA*, and *TLR4* expression^4,11^. This urea concentration is normally found in healthy cows fed with high-protein diet (17 to 19%)^55,56^ and causes reduced fertility^57^. This implied that the high level of urea or LPS alleviated the immune system in the oviduct *via* inducing an anti-inflammatory environment, predisposing cows to infections.

The expression of *IL1B*, *TNFA*, *IL10* (negatively correlated, under pathophysiological conditions) and *IL10* (negatively correlated, under pathophysiological + physiological conditions) provided the best prediction for *IL4* expression. Furthermore, the co-expression of *IL1B* and *IL10* provided a good prediction for *IL4* expression under all experimental conditions. Previous studies have reported that IL4 may exhibit both pro- and anti-inflammatory properties, for example, the over-expression of *Il4* in case of colitis in mice intestine^19^; in another study, it was reported that IL4 inhibits the production of IFN-γ by IL12-exposed CD8T cells^58^. Due to the negative relationship between *IL4* and other pro-/anti-inflammatory cytokine genes (i.e., *IL1B*, *TNFA*, or *IL10*) observed in this study, we cannot conclude the exact function of *IL4* (anti- versus pro-inflammatory function) during LPS or toxin challenges in BOECs. This issue should be considered in future experiments.

The co-expression of *TNFA* and *IL4* under physiological conditions, the co-expression of *TNFA* and *IL1B* under pathophysiological conditions, or the co-expression of *TNFA* and *TLR4* under pathophysiological + physiological conditions provided the best prediction for *IL10* expression (Fig. 5d). These findings revealed a hidden relationship among gene pairs under different experimental conditions. Data showed that *TNFA* (either alone or in co-expression with other genes) was expressed in parallel with *IL10*, suggesting a role for *IL10* expression in controlling the expression of pro-inflammatory *TNFA*^49^ under pathophysiological + physiological conditions.

Collectively, in the present study, we predicted the mRNA expression of target genes using the data from other genes and identified the hidden relations among them using a highly nonlinear polynomial model (a multi-layer response surface method). The data collected confirmed an alteration in the relationship between genes based on the experimental conditions and the co-expression of other gene pairs. Moreover, data revealed that the presence of the pre-ovulatory ovarian sex steroids, LH, and sperm cells mainly regulated the co-expression of anti- and pro-inflammatory cytokine genes, resulting in an optimal environment for the early reproductive processes. As a limitation of this study, due to a lack of data availability, we excluded the genes with insufficient data points and thus narrowed the small number of genes (five genes). Therefore, it should be noted that, when a greater number of genes is investigated, the observed relationship between genes will definitely change. Finally, these results may help the user in designing next experiments and (if applicable) in deciding the specific drugs to apply based on the gene-gene relationship and various biological conditions of patients.

## Methodology

Animal experiments were carried out according to the Guiding Principles for the Care and Use of Research Animals promulgated by the Obihiro University of Agriculture and Veterinary Medicine, Japan. The protocols and methods were approved by the Committee on the Ethics of Animal Experiments of the Obihiro University of Agriculture and Veterinary Medicine (permit number 25–101).

The data were obtained from 16 independent *in vitro* experiments, BOECs culture, with four to five replications (including published and unpublished data) that were conducted by our group in Japan (Table 3). As shown in the Supplementary text (see Supplementary Table S1), *IL10*, *TNFA*, *IL1B*, and *IL4* cytokine genes had the highest skewed distribution, respectively. This implied that the data for the mRNA expression had a non-normal distribution. The X_max_ to X_min_ ratios of all genes had values greater than 1000. So, in order to reduce the interval domain, data were computed using the logarithmic basis function.

### Isolation and culture of bovine oviduct epithelial cells (BOECs)

First, in the local slaughterhouse, Hokkaido (Japan), we macroscopically examined the female reproductive tracts (FRT) and selected the healthy ones (free of any cystic follicle, swelling, abscesses, inflammation, pus, and abnormal color).The selected oviducts were immersed in phosphate-buffered saline (PBS) solution without Ca^2+^/Mg^2+^ (PBS^−/−^) (Sigma, St. Louis, MO, USA) containing 0.3% gentamicin (Sigma) and amphotericin B (Illkirch Graffenstaden, France) and thereafter transported to the laboratory using in an ice box. Before the epithelial cells isolation, we determined the stage of the ovarian cycle and examined oviducts, then selected those free of any inflammation, swelling, abscesses, and pus. For all BOECs experiments, the lumen of the pre-ovulatory oviducts (day 19–20 of the cycle) was solely flushed using PBS (15 mL) and mechanically dislodged with the same volume of PBS. Therefore, it was assumed that the epithelial cells used for all independent experiments including control group were isolated from a similar hormonal environment (the pre-ovulatory phase). Also, since the sampling was carried out at different times (between the years 2010 and 2016), the oviducts were collected under different conditions (i.e., a cold *vs*. hot weather) or derived from cows with different diets. Thus, these factors could cause different responses in the control group. However, in order to reduce the individual variation, the epithelial cells from four to five cows were pooled as a biological replication. The pre-ovulatory stage was determined by the existence of at least one follicle greater than 10 to 15 mm in diameter and a regressing corpus luteum (<1 cm in diameter), firm in consistency and without vasculature visible on its surface according to previous reports^59,60^.

To collect epithelial cells, the cells from four to five oviducts were pooled and allowed to settle down at the bottom of the tubes for 15 min. The settled cells were washed with PBS and then with a medium containing D-MEM/F12, 0.1% gentamicin, 1% amphotericin and 2.2% NaHCO_3_. Next, we harvested cells, as a pellet, by centrifugation at 300 × *g* for 10 min at 4 °C and suspended in 10 mL of PBS. At this step, the cells were layered over with 10 mL Percol and centrifuged at 900 × *g* for 20 min at 4 °C. Next, we harvested cells by centrifugation at 300 × *g* for 10 min at 4 °C and cultured them in the 6-well culture dishes (Nalge Nunc International, DK-4000 Roskilde, Denmark) containing the culture medium (D-MEM/F12, 0.1% gentamicin, 1% amphotericin, 2.2% NaHCO_3_, and 10% fetal calf serum [FCS; BioWhittaker, Walkersville, MD, USA]) at 38.5 °C in 5% CO_2_ and 95% air. On the following day, we washed the cultures twice with PBS and cells were again placed in 6-well culture dishes at a density of 3 × 10^4^/mL and incubated at 38.5 °C in 5% CO_2_ and 95% air in culture medium supplemented with 5% FCS. The medium was renewed every 48 h until the growing BOECs monolayer covered about 70–80% of the bottom of the culture plate. When the cultured cells formed a monolayer, we trypsinized cells using 0.05% trypsin EDTA (Amresco, Solon, OH, USA) until single cells appeared.

During the cell cultures, we stimulated BOECs at the first passage. Since the BOECs cultures with a high passage number show lower cell viability and change the pro-inflammatory responses^61^. Also, because of different sensitivities of cultured cells to trypsin during the passage process, some cells may remain permanently adhered to the plate. This may result in non-constant and divergent gene expression pattern in the passages later^62^. Danesh Mesgaran *et al*.^63^ reported that the number of cell culture passage can change the mRNA expressions of genes. Also, they found that BOECs at passage 3 secreted more IL8 compared with those in the passage zero^63^. Hirth *et al*.^64^ reported that in a repeated-passage culture of rat fibroblast, the expression of *Il10* was reduced. They also found a reduced TNFA secretion while the mRNA expression of *Tnfa* was increased. They suggested that the early passage cells gave a better source of cells which have *in vivo* phenotype/functional features^64^. Compared to these findings, we had stimulated BOECs cultures in the first passage.

In the first passage, the cells were cultured until they covered about 70–80% of the bottom of the culture plate. Then, BOECs cultures were washed twice using the culture medium supplemented with 0.1% FCS and incubated for 24 h with varying concentrations of (1) LPS (*E. coli* serotype 055: B5; Sigma) (1, 10, 100, or 1000 ng/mL); (2) bovine AGP (alpha-1-acid glycoprotein or orosomucoid, Life Diagnostics, Inc., West Chester, PA, USA) (1, 10, 100, or 1000 ng/mL); (3) zearalenone (MP Biomedicals, Germany) (1, 10, 100, or 1000 ng/mL); (4) urea (Sigma) (20, 40, or 80 mg/dL); (5) LPS (10 or 100 ng/mL) + AGP (10 or 100 ng/mL); (6) luteinizing hormone (LH, USDA-bLH-B6, USDA Animal Hormone Program, Bethesda, MD, USA) (10 or 20 ng/mL); (7) estradiol (E2, sigma) (1 ng/mL); (8) progesterone (P4, sigma) (1 ng/mL); (9) a combination of sex hormones (ovarian sex steroids and luteinizing hormone; E2 + P4 + LH; 1, 1, and 10 ng/mL, respectively); (10) PGE2 (sigma) (3.52, 35.2, or 352 ng/mL); (11) sperm cells (2 × 10^5^ sperm/mL); (12) LPS (10 or 100 ng/mL) + E2, LH, or P4 (1, 10, or 1 ng/mL, respectively); (13) LPS (10 or 100 ng/mL) + E2 + P4 + LH (1, 1, and 10 ng/mL, respectively); (14) zearalenone (1, 10, or 100 ng/mL) + E2 + P4 + LH (1, 1, and 10 ng/mL, respectively); (15) zearalenone (1, 10, 100, or 1000 ng/mL) + co-culture of BOECs with sperm cells; (16) urea (20 or 40 mg/dL) + E2 + LH + P4 (1, 10, or 1 ng/mL, respectively). In parallel with each of the independent experiments, BOECs were cultured in the medium in the absence of stimulants and classified as the basic class (control).

In general, based on the incubation with different stimulants, BOECs were classified into four classes, including (1) the basic class (BOECs were incubated with no stimulants, control.); (2) the physiological class (BOECs were incubated with sperm cells, the pre-ovulatory levels of sex hormones, such as E2, P4, and LH, or PGE2.); (3) the pathophysiological class (BOECs were incubated with toxins, such as LPS, zearalenone, and urea, or with AGP as an infection-related protein.); and (4) the pathophysiological + physiological class (the combination of LPS and sex hormones, zearalenone and sex hormones, or urea and sex hormones).

The basic class describes the conditions at which BOECs were solely incubated with culture medium. In order to determine the relationships between genes under normal/healthy conditions with no interference from LPS or toxins, the physiological class describes the conditions at which BOECs were incubated with physiological components, such as sex hormones (LH, E2, or P4 at the pre-ovulatory levels), PGE2 or sperm cells, under normal circumstances. For example, it has been experimentally reported that in response to live sperm cells, BOECs secret PGE2^2,3^ which, in turn, could favor a pro-inflammatory response^3^. Thus, the conditions at which BOECs were stimulated with PGE2 were grouped into the physiological class. This class was designed to help us identify the relationship between the mRNA expressions of both pro- and anti-inflammatory cytokine genes under normal conditions.

To identify the relationship between pro- and anti-inflammatory cytokine genes during abnormal situations, the pathophysiological class was designed; it describes the conditions at which BOECs were incubated with LPS, AGP (infection-related protein) or toxins (such as zearalenone or urea). 10 ng/mL of LPS has been reported to induce a pro-inflammatory response (up-regulation of *IL1B*, *TNFA*, *TLR4*, and *NFKBIA*) in BOECs, while a higher dose of LPS (100 ng/mL) induced the mRNA expression of *IL10* and *IL4* and reduced *NFKBIA*, *TLR4*, *IL1B*, and *TNFA* expression^11^. These physiological changes caught our attention that the mRNA expression of these pro- and anti-inflammatory cytokine genes could trigger each other during abnormal conditions. Furthermore, both pathophysiological and physiological classes were utilized to interpret the changes in the gene-gene relationship by comparing gene expressions under these conditions.

Also, the physiological components, such as ovarian sex steroid hormones, LH, or sperm cells, may alter the immune response caused by LPS or toxins, and vice verse. For example, Kowsar *et al*.^11^ and Fahey *et al*.^1^ reported that ovarian sex steroids inhibited LPS-induced expression of pro-inflammatory mediators. It has been shown that zearalenone abrogated the anti-inflammatory responses in co-culture of sperm cells and BOECs^10^. Hence, the pathophysiological + physiological class was designed to identify how the associations among genes are altered in the presence of both physiological and pathophysiological components.

In all BOECs experiments, the pre-ovulatory levels of the ovarian steroid hormones and LH were used according to previous studies^65,66^. The concentrations of urea and PGE2 were based on previous studies^2,67,68^. Also, sperm preparation and co-culture of BOECs with swim-up sperm cells (2 × 10^5^ sperm/mL) were described in our earlier work^3^. We tested the purity of epithelial cells using the reaction of the cultured cells with the monoclonal antibodies to cytokeratin (anti-cytokeratin-CK1) and immunostaining. The cells in the culture medium showed characteristic epithelial morphology, and 98% of the cells were positive for anti-cytokeratin (CK1) antibodies. We evaluated the cell viability using Trypan blue staining at each plating time and the end of each experiment. All BOECs cultures presented more than 90% cell viability which was consistent with the report of Rottmayer *et al*.^69^ that after a 24h-culture, BOECs still showed 95% purity and maintained *in vivo* morphological characteristics. Before each independent experiment, we performed preliminary dose-response studies to understand the potential of the toxins applied during the 24h-incubation period. We discovered that dose of LPS at 1000 ng/mL^11^, urea at 80 mg/dL^4^, and zearalenone at 1000 ng/mL were toxic and reduced the epithelial cell viability after 24 h. Thus, data from these toxic concentrations were excluded from the analysis. All data were produced from 16 independent experiments (BOECs cultures) each with four to five replications. All BOECs (conducted between the years 2010 and 2016) were isolated from the oviducts in the pre-ovulatory phase (day 19–20 of the cycle), incubated (after first passage) for 24 h with the above-mentioned stimulants using the same experimental conditions and same protocols.

### Extraction of RNA, production of cDNA, and real-time polymerase chain reaction (real-time PCR)

After 24 h stimulation, we collected epithelial cells and extracted RNA from the cells using TRIzol (Invitrogen). The quantity of the extracted RNA was assessed using an ultraviolet (UV) spectroscopy (optical density, 260). Using a spectrophotometer (Eppendorf, Munich, Germany), the ratio of the absorbance at 260 and 280 nm (A260/280) was used to measure the purity of the RNA. The extracted RNA was kept in RNA storage solution (Ambion, Austin, TX, USA) at −80 °C until cDNA production. DNase treatment was carried out using the RQ1 RNase-Free DNase kit (Promega, Madison, WI, USA). The synthesized cDNA was stored at −30 °C. Using qRT-PCR, we quantified the mRNA expression of *IL1B*, *TNFA*, *IL10*, *IL4*, and *TLR4*. The quantifications of mRNA expression were done using synthesized cDNA *via* real-time PCR on a LightCycler (Roche Diagnostics, Mannheim, Germany) with a QuantiTect™ SYBR Green PCR Master Mix (QIAGEN, Hilden, Germany). The amplification program comprised 15 min of activation at 95 °C, followed by 40 cycles of PCR (15 sec of denaturation at 95 °C, 30 sec of annealing at 54 to 58 °C and 20 sec of extension at 72 °C). We normalized the values of mRNA expression related to β-actin (*ACTB*) as the internal standard. The expressions of *ACTB* were stable in all experiments and showed no significant differences between treatments. The specific primers were designed based on the bovine sequences using PRIMER EXPRESS software (Perkin-Elmer, Boston, MA) as shown in Supplementary text (Supplementary Table S2).

### Methods for detecting the relations among genes (modeling approaches)

In the present study, the mathematical modeling is referred to the relations among genes. The regression procedure is defined using the following relation (Eq. 1):

1 $$G=f({\boldsymbol{x}})+\varepsilon $$

where *G* is the observed gene expression data. *f*(***x***) is the calibrated function that regressed based on the input variable vector ***x***. *ε* is the error term. Also, we considered three different mathematical forms to state the calibrated function *f*(***x***). These three approaches were described in the following parts.

### Multiple-linear regression (MLR)

A simple linear mathematical form (first-order polynomial function) was applied into the MLR using the following calibrated function (Eq. 2):

2 $$f({\boldsymbol{x}})={\beta }_{0}+\sum _{i=1}^{n}{\beta }_{i}\,{x}_{i}$$

where ${\beta }_{i}\,i=0,\,1,\,\mathrm{...},\,n$ are the unknown coefficients and *n* is the number of the input variables.

### Response surface method (RSM)

The RSM calibrates the gene expression data based on the second-order polynomial basis function (Eq. 3)^70^:

3 $$f({\boldsymbol{x}})={\beta }_{0}+\sum _{i=1}^{n}{\beta }_{i}{x}_{i}+\sum _{i=1}^{n}\sum _{j\ge i}^{n}{\beta }_{ij}{x}_{i}{x}_{j}$$

where *β*_0_, *β*_*i*_, and *β*_*ij*_, are the unknown coefficients of the polynomial function with cross terms, *f*(***x***).

### Multi-layer response surface method (MLRSM)

In order to improve the accuracy and flexibility of the model in defining a suitable relationship among genes, we developed the MLRSM model based on the multilayer strategy and the number of the input dataset as scenario 1: the mRNA expression of one gene was used as the input dataset for predicting the output gene (Fig. 1a), scenario 2: the mRNA expression of two genes (gene pair) was used as the input dataset for predicting the output gene (Figs 1b,d and 2), and scenario 3: the mRNA expression data of three genes was used as the input dataset for predicting the output gene (Fig. 1c). A detail of the models is provided in the Supplementary text.

Based on the results obtained, the MLRSM scenario 2 was observed to outperform other models and scenarios (Table 1) thus only this model was described in detail (Figs 1b,d and 2, and Supplementary text). A representative structure of the MLRSM scenario 2 (i.e., the prediction of *IL1B* mRNA expression) was described in detail in Fig. 2. As seen, the MLRSM scenario 2 was structured using two main calibrating processes (Figs 1d and 2). In the first stage, the high-nonlinear polynomial function was employed to calibrate the hidden database (predicted gene pairs) (*y)* by using the input dataset (*x*), such as *TNFA*, *IL4*, *TLR4*, and *IL10*. The hidden database (layer) was shown as the predicted gene pairs in Figs 1d and 2a. Next, the handling predicted data (the hidden layer, *y*) obtained from the first calibrating process were employed for the regression analysis of the target (output) gene (*G*) (i.e., *IL1B*) in the second stage (Figs 1d and 2b).

Because of including the hidden layer in the first modeling approach, the model obtained the ability to consider the correlation between the input genes (*x*). Thus, the data of the hidden layer (predicted gene pairs) of this model was utilized to construct Fig. 5a–e (the relationship between gene pairs and the output gene as seen in Fig. 2c).

Each dataset (*NS*) of the hidden layer can be calibrated from the input dataset using the high-order polynomial functions (Eq. 4).

4 $${y}_{l}={\beta }_{0}+\sum _{i=1}^{NS}{\beta }_{i}{x}_{i}+\sum _{i=1}^{NS}\sum _{j=i}^{NS}{\beta }_{ij}{x}_{i}{x}_{j}+\sum _{i=1}^{NS}\sum _{j=1}^{NS}{c}_{ij}{x}_{i}{x}_{j}^{2}+\sum _{i=1}^{NS}\sum _{j=1}^{NS}{d}_{ij}{x}_{i}{x}_{j}^{3}+\sum _{i=1}^{NS}\sum _{j=1}^{NS}{e}_{ij}{x}_{i}{x}_{j}^{4}$$

where *β*_0_, *β*_*i*_, *β*_*ij*_, *c*_*ij*_, *d*_*ij*_ and *e*_*ij*_ are the unknown coefficients. *y*_*l*_ is the handling calibrated data of the *l*_th_ element of the hidden layer with a total of *M*-element in the hidden layer, i.e., *l* = 1, 2, …, *M* that is given based on the nonlinear forms using the input gene dataset. The input dataset of genes for each element of the hidden layer should be selected but to ${y}_{i}\ne {y}_{j}$.

Finally, using the following high-order polynomial functions (Eq. 5), we estimated the calibrated results of the output (target) gene (*G*).

5 $$f({\boldsymbol{x}})={\beta ^{\prime} }_{0}+\sum _{i=1}^{M}{\beta ^{\prime} }_{i}{y}_{i}+\sum _{i=1}^{M}\sum _{j=i}^{M}{\beta ^{\prime} }_{ij}{y}_{i}{y}_{j}+\sum _{i=1}^{M}\sum _{j=1}^{M}{c^{\prime} }_{ij}{y}_{i}{y}_{j}^{2}+\sum _{i=1}^{M}\sum _{j=1}^{M}{d^{\prime} }_{ij}{y}_{i}{y}_{j}^{3}+\sum _{i=1}^{M}\sum _{j=1}^{M}{e^{\prime} }_{ij}{y}_{i}{y}_{j}^{4}$$

In general, the nonlinear form can be utilized in two calibrated steps (i.e., in the hidden layer and then in the calibrated results, the output gene). The two-step process (Figs 1d and 2a,b) and the higher-order polynomial basis functions (Eq. 5) are two major differences that we can mention between the MLRSM model and other models (RSM and MLR).

We implemented our approach (MLRSM scenario 2) in MATLAB using approximately 120 lines of code (Supplementary text). The details of the calibration procedure are outlined in the Supplementary text. A significant relationship that existed (P < 0.05) between genes was determined using Akaike Information Criteria (*AIC*) as described before^26^ (Supplementary text).

### Comparative statistics and calculation of Pearson’s rank correlation

Several statistical comparative factors were applied to test the accuracy of prediction for the genes based on the MLR, RSM, and MLRSM models. These comparative statistics^70–72^ were:

*Root-mean-square error (RMSE)*

6 $$RMSE=\sqrt{\frac{1}{N}\sum _{i=1}^{N}{[{({G}_{o})}_{i}-{({G}_{p})}_{i}]}^{2}}$$

*Mean bias error (MBE)*

7 $$MBE=\frac{1}{N}\sum _{i=1}^{N}\frac{{({G}_{o})}_{i}-{({G}_{p})}_{i}}{{({G}_{o})}_{i}}$$

*Model efficiency factor (EF)*

8 $$EF=1-\frac{\sum _{i=1}^{N}{[{({G}_{o})}_{i}-{({G}_{p})}_{i}]}^{2}}{\sum _{i=1}^{N}{[{({G}_{o})}_{i}-{\bar{G}}_{o}]}^{2}},\,-\,\infty  < EF\le 1$$

*Agreement index (d)*

9 $$d=1-\frac{\sum _{i=1}^{N}{[{({G}_{o})}_{i}-{({G}_{p})}_{i}]}^{2}}{\sum _{i=1}^{N}{[|{({G}_{o})}_{i}-{\bar{G}}_{o}|+|{({G}_{p})}_{i}-{\bar{G}}_{o}|]}^{2}},\,0 < d\le 1$$

where *N* is the number of experimental data. *G*_*o*_ and *G*_*p*_ are respectively the experimental and predicted data points for a gene using the data from other genes as input data. ${\bar{G}}_{o}$ is the average value of the observed genes determined by ${\bar{G}}_{o}=\frac{1}{N}\sum _{i=1}^{N}{({G}_{o})}_{i}$. *RMSE* shows the average difference between the predicted (*G*_*p*_) and observed (*G*_*o*_) gene for *i*_*th*_ data. Reduced values of *RMSE* and *MBE* show a better prediction. Efficiency factor (*EF*) shows the correlation between the predicted and experimental data and shows the goodness-of-fit of the model between prediction and observations and R^2^. The agreement in*d*ex, *d*, can be implemented to make a cross-comparison between the predicted and experimental data ranging from 0 (no correlation) to 1 (perfect correlation) and R^2^ ^73^. Based on the accuracy of the model’s prediction, *d* is better suited than R^2^ for the model evaluation. To test the direction (a negative or a positive correlation) of the relationship among genes, we performed the correlation analysis using the Pearson’s rank correlation analysis method^73^.

### Nonlinear-based principal components analysis (PCA) method

The nonlinear-based principal components analysis (nonlinear PCA) transforms several correlated variables into a smaller number of uncorrelated variables^25^. This method simplifies the complexity of high-dimensional data^74^. PCA determines the patterns of datasets with a high dimension. The main advantage of PCA is to find other patterns for datasets and compresses the data of genes by reducing their number of dimensions. Nonlinear PCA compresses the expression data of genes with five dimensions into two dimensions. Then, PCA illustrates the relations between the predicted and experimental data. Therefore by using the nonlinear-based PCA, we reduced the dimensions of mRNA expression data of genes in both experimental and predicted phases and evaluated the prediction accuracy of MLRSM model^75^.

## Supplementary information

Understanding the hidden relations between pro- and anti-inflammatory cytokine genes in bovine oviduct epithelium using a multilayer response surface method

## Data Availability

The datasets of the present study are available from the corresponding authors upon reasonable requests.

**Table 3:** The independent bovine oviduct epithelial cell cultures (BOECs) were experimented between the years 2010 and 2016, reporting the effects of various stimulants on the mRNA expression of the pro- versus anti-inflammatory cytokine genes.

Stimulants	Dose	Reference
**The pathophysiological class (n = 230 data)**		
Lipopolysaccharide (LPS)	1, 10, 100, 1000 ng/mL	Kowsar *et al*.^11^
Alpha-1-acid glycoprotein (AGP)	1, 10, 100, 1000 ng/mL	Kowsar *et al*.^13^
Zearalenone	1, 10, 100, or 1000 ng/mL	Two independent experiments (unpublished and Yousef *et al*.^10^)
Urea	20, 40, or 80 mg/dL	Kowsar *et al*.^4^
LPS + AGP	(10, 100 ng/mL) + (10,100 ng/mL)	Kowsar *et al*.^13^
**The physiological class (n = 270 data)**		
Luteinizing hormone (LH)	10 or 20 ng/mL	Unpublished
Estradiol (E2)	(1 ng/mL)	Unpublished
Progesterone (P4)	(1 ng/mL)	Unpublished
LH + E2 + P4	1, 1, 10 ng/mL, respectively	Unpublished
Prostaglandin E2 (PGE2)	3.52, 35.2, or 352 ng/mL	Two independent experiments (unpublished and Yousef *et al*.^3^)
Sperm cells	2 × 10^5^ sperm/mL	Yousef *et al*.^3^
**The pathophysiological + physiological class (n = 215 data)**		
LPS + E2, LH, or P4	(10 or 100 ng/mL) + 1, 10, or 1 ng/mL, respectively.	Kowsar *et al*.^11^
LPS + E2 + LH + P4	(10 or 100 ng/mL) + 1 + 10 + 1 ng/mL, respectively.	Unpublished
Zearalenone + E2 + LH + P4	(1, 10, 100 ng/mL) + 1 + 10 + 1 ng/mL, respectively.	Unpublished
Zearalenone-treated BOECs + sperm cells	(1, 10, 100, or 1000 ng/mL) + 2 × 10^5^ sperm/mL	Yousef *et al*.^10^
Urea + E2 + LH + P4	(20 and 40) + 1 + 10 + 1 ng/mL, respectively.	Unpublished
**Control, BOECs incubation with no stimulants (n = 95 data)**		

Data were produced from 16 independent experiments each with four to five replications. All BOECs cultures were incubated for 24 h with the above-mentioned stimulants following the same experimental conditions and same protocols. In the case where BOECs were incubated with sex hormones, the pre-ovulatory levels of hormones were used according to the previous studies^68,69^. The concentrations of urea, sperm cells, and PGE2 in the present research were based on the previous studies^2,3,70,71^. Before each independent experiment, the preliminary dose-response studies were performed to understand the potential of the applied toxins after 24 h incubation; high doses, such as LPS at 1000 ng/mL^11^, urea at 80 mg/dL^4^, and zearalenone at 1000 ng/mL, were toxic and reduced the epithelial cell viability. Thus, data from these toxic concentrations were excluded from the analysis. The independent bovine oviduct epithelial cell cultures (BOECs) were experimented between the years 2010 and 2016, reporting the effects of various stimulants on the mRNA expression of the pro- versus anti-inflammatory cytokine genes. Data were produced from 16 independent experiments each with four to five replications. All BOECs cultures were incubated for 24 h with the above-mentioned stimulants following the same experimental conditions and same protocols. In the case where BOECs were incubated with sex hormones, the pre-ovulatory levels of hormones were used according to the previous studies^68,69^. The concentrations of urea, sperm cells, and PGE2 in the present research were based on the previous studies^2,3,70,71^. Before each independent experiment, the preliminary dose-response studies were performed to understand the potential of the applied toxins after 24 h incubation; high doses, such as LPS at 1000 ng/mL^11^, urea at 80 mg/dL^4^, and zearalenone at 1000 ng/mL, were toxic and reduced the epithelial cell viability. Thus, data from these toxic concentrations were excluded from the analysis.
