# Supplementary material for: Understanding the hidden relations between pro- and anti-inflammatory cytokine genes in bovine oviduct epithelium using a multilayer response surface method
Source: Sci Rep. 2019 Feb 28;9:3189. doi: 10.1038/s41598-019-39081-w (PMC6395797; doi:10.1038/s41598-019-39081-w)
Supplement: Supplementary file 1 — Understanding the hidden relations between pro- and anti-inflammatory cytokine genes in bovine oviduct epithelium using a multilayer response surface method [file 41598_2019_39081_MOESM1_ESM.pdf]

Understanding the hidden relations between pro- and anti-inflammatory cytokine genes in bovine oviduct epithelium using a multilayer response surface method

Rasoul Kowsar[1, 2*], Behrooz Keshtegar[3*], Akio Miyamoto[2]

[1]Department of Animal Science, College of Agriculture, Isfahan University of Technology, Isfahan, 84156–83111, Iran.

[2]Graduate School of Animal and Food Hygiene, Obihiro University of Agriculture and Veterinary Medicine, Obihiro, Hokkaido 080-8555, Japan.

[3]Department of Civil Engineering, Faculty of Engineering, University of Zabol, P.B. 9861335-856, Zabol, Iran.

* **To whom correspondence should be addressed**: Rasoul_kowsarzar@yahoo.com; bkeshtegar@yahoo.com

Contents:

1. **Supplementary Results**
2. **Supplementary Discussion**
3. **Supplementary Methods**
   3.1 The detailed description of the models.
   3.2 The procedure of the calibration.
   3.3 Akaike Information Criteria (*AIC*)
   3.4 User-friendly codes employed for MLRSM scenario 2 in MATLAB.
   3.5 Supplementary Table S1-S2

1. Supplementary Results

The non-significant relationship among the candidate genes predicted by Akaike Information Criterion (*AIC*). Akaike Information Criterion (*AIC*) discovered no significant relationship ($P > 0.05$) between the mRNA expression of *IL1B* and that of *IL10* and *IL4* under basic or pathophysiological conditions. Also, under physiological or pathophysiological + physiological conditions, the expression data of *IL1B* had no significant relationship ($P > 0.05$) with *TLR4* in BOECs culture (**Table 2**).

Using *AIC* analysis, we identified no significant relationship ($P > 0.05$) between *TNFA* mRNA expression and (1) *TLR4* mRNA expression under all experimental conditions and (2) *IL4* expression under physiological, pathophysiological or pathophysiological + physiological conditions (**Table 2**).

AIC analysis showed no significant association ($P > 0.05$) between *TLR4* mRNA expression and (1) *IL10* under all experimental conditions; (2) *IL4* under basic, pathophysiological, or pathophysiological + physiological conditions; and (3) *TNFA*, *IL4*, and *IL10* expression under pathophysiological conditions.

The *AIC* revealed that the mRNA expression data of *IL4* had no significant relationship ($P > 0.05$) with *TNFA* expression under basic conditions or *IL10* expression under physiological conditions. There was no significant association between *IL4* and *TLR4* mRNA expression under pathophysiological or pathophysiological + physiological conditions (**Table 2**).

The *AIC* showed no significant relationships ($P > 0.05$) between the expression data of *IL10* and *IL4* or *TLR4* under physiological, pathophysiological, or pathophysiological + physiological conditions (**Table 2**).

2. Supplementary Discussion

In the present study, we investigated one linear (MLR) and two different nonlinear models (RSM and MLRSM) to determine the best model that could fit the experimental data. Then, using the best-observed model, we sought to find the gene that its expression data provided the best prediction for the expression

of other genes under various experimental conditions. We also examined the relationship between the mRNA expression of a gene and the co-expression of other gene pairs.

The mathematical form of response surface (RSM) function is also simple. But, the second-order terms of the input variables in this model may give more exact results, compared to the MLR. Because of the higher-order relations among genes, the linear models, such as MLR, showed just the main effects of genes but could not detect the nonlinearity among the genes[1]. Also, the MLR does not cater for multicollinearity problem (multiple correlations)[2,3]. If the independent variables show a high correlation with the main response and with each other, then multicollinearity problems will occur[2]. The RSM model as a least-squares modeling-based technique considers the quadratic (or cubic) effects and complex interactions between gene pairs[1]. So, the RSM can reduce the multicollinearity problem (based on the present ordinary least squares (OLS) estimator that uses the transformed data as used in this study)[3,4,5]. Thus, besides detecting the independent effect of variables over a response, RSM can detect the relations among the variables[5,6].

We discovered that, the scenario 2 of MLRSM model had the highest correlation (*d)* and goodness-of-fit (*EF*) and the lowest error (*RMSE*), compared to the MLR, RSM and MLRSM (scenarios 1 to 3). Also, the scatterplots of the predicted and experimental data confirmed a strong nonlinear relationship among the candidate genes. Considering the scatterplots, scenario 2 showed a better prediction and evaluated a proper coefficient for the input data points. Thus, the MLRSM scenario 2 outperformed the MLR, RSM, scenarios 1 and 3 of MLRSM in showing prediction accuracy. These findings suggested the MLRSM scenario 2 was a robust model for detecting high-order nonlinear relationships among genes. Scientific data show both linear and nonlinear responses to the physiological or non-physiological conditions in biological events, such as gene-gene interactions[7,8,9]. Solvang *et al*.[10] reported a linear relation among genes related to the metabolism. But, they found a nonlinear relationship among genes involved in the immune system (for example, IL2 signaling)[10]. Similarly, our results showed a strong nonlinearity among the present immune-related genes. To the best of our knowledge, the

MLRSM model was used for the first time to detect the relations among genes. However, several previous studies have applied the RSM model to examine the changes in gene expression under experimental conditions[11]. For example, Howard *et al*.[12] used the RSM model to determine gene-gene relationship existing among the quantitative trait loci (QTLs).

Next, in order to evaluate the prediction accuracy of the MLRSM scenario 2 as well as to explore the main gene expression pattern, we input the scenario 2-predicted data into the nonlinear-based PCA[13]. The nonlinear PCA showed a good association between each predicted data point and corresponding actual data point of *IL10*, *IL4*, *TLR4*, *IL1B*, and *TNFA* (*RMSE* values were low and ranged from 0.004 to 0.209 for these genes). Also, the nonlinear PCA showed that the mRNA expression of candidate genes under experimental conditions (physiological, pathophysiological, or pathophysiological + physiological conditions) was similarly predicted by the predictor (the MLRSM scenario 2). This suggested the similarity of the gene expression patterns within samples obtained from the same experimental conditions, implying a proper preparation and choice of BOECs samples[14].

3. Supplementary Methods

3.1 The detailed description of the models.

Multiple-linear regression (MLR). To make this model easy to understand, we explain the model with three input variables. In the following equation (**Supplementary Eq. S1**), g_1, to g_3 are the gene expression data of genes (input variables), for example, *IL10*, *IL4*, and *TNFA.* The output or predicted gene (*G*), such as *IL1B*, can be estimated in a linear format using the MLR model (**Supplementary Eq. S1**):

$$G = \beta_0 + \beta_1 g_1 + \beta_2 g_2 + \beta_3 g_3 \qquad \text{(S1)}$$

where β_0 to β_3 are the unknown coefficients.

Response surface method (RSM). To make the RSM model easy to understand, we explain this model with three input variables. As, in the following equation (**Supplementary Eq. S2**), $g_{1,}$ to g_3 are the mRNA expression data (input variables), for example, *IL10*, *IL4*, and *TNFA*. The output or predicted target gene (G), such as *IL1B*, can be estimated in a nonlinear format using the RSM model (**Supplementary** Eq. S2):

$$G = \beta_0 + \beta_1 g_1 + \beta_2 g_2 + \beta_3 g_3 + \beta_{11} g_1^2 + \beta_{12} g_1 g_2 + \beta_{13} g_1 g_3 + \beta_{22} g_2^2 + \beta_{23} g_2 g_3 + \beta_{33} g_3^2 \qquad \text{(S2)}$$

Using the second-order basis polynomial function in the RSM model (**Supplementary Eq. S2**), we can, therefore, improve the nonlinear relations in predicting complex problems, i.e., the predicted target gene (G). Compared with the linear relation (**Supplementary Eq. S1**), the nonlinear formulation-based second-order polynomial function (**Supplementary Eq. S2**) can provide a proper relationship for estimating the predicted target gene (G). Because the RSM model comprised a one-step process, the model forms a general relation between the input (g) and output (target gene) variables (G)[15]. This infers the limits of RSM in dealing with many correlated responses and in estimating the prediction accuracy[16].

Multi-layer response surface method (MLRSM). To make this model (scenario 2) easy to understand, we explain the two-step process of this model with three input variables. In the following equation (**Supplementary Eq. S3**), g_1 to g_3 were the expression data of input genes, for example, *IL10*, *IL4*, and *TNFA* (**Note***:* in **Fig. 1d**, the input genes were shown as x_1 to x_n). As mentioned before, the model comprised two calibration processes. At the first, we calibrated the hidden layer (y_1 to y_3) of the model using the input genes (x_1 *to* $x_{3,}$ **Fig. 1d)** which are equivalent to g_1 to g_3 in **Supplementary Eq. S3** to **Supplementary Eq. S5**. The total number of the elements in the hidden layer (M) was calculated based on the number of input genes (NS) and a total number of genes (n) as $M = \frac{n!}{(n-NS)!NS!}$ where ! is the factorial operator and $NS \leq n$. For example, we calculated M = 4 for the scenario 1 where n = 4 and NS =

1 (i.e., $M = \frac{n!}{(n-NS)!NS!} = \frac{4!}{(3)!1!} = 4$); M = 6 for the scenario 2 where n = 4 and NS = 2; and M = 4 for the scenario 3 where n = 4 and NS = 3.

The first calibrating process was employed to predict the hidden layer elements (y) (**Fig. 1d and Fig. 2a**). So, using the following nonlinear relations (**Supplementary Eq. S3** to **Supplementary Eq. S5**) and the input datasets (g_1 to g_3), we first estimated each element of the hidden layer (y) in the scenario 2 of MLRSM.

$$\begin{aligned} y_1 = {} & \beta_0 + \beta_1 g_1 + \beta_2 g_2 + \beta_{11} g_1^2 + \beta_{12} g_1 g_2 + \beta_{22} g_2^2 + c_{11} g_1^3 + c_{12} g_1 g_2^2 + c_{21} g_2 g_1^2 + c_{22} g_2^3 \\ & + d_{11} g_1^4 + d_{12} g_1 g_2^3 + d_{21} g_2 g_1^3 + d_{22} g_2^4 + e_{11} g_1^5 + e_{12} g_1 g_2^4 + e_{21} g_2 g_1^4 + e_{22} g_2^5 \end{aligned} \tag{S3}$$

$$\begin{aligned} y_2 = {} & \beta_0 + \beta_1 g_1 + \beta_2 g_3 + \beta_{11} g_1^2 + \beta_{12} g_1 g_3 + \beta_{22} g_3^2 + c_{11} g_1^3 + c_{12} g_1 g_3^2 + c_{21} g_2 g_1^2 + c_{22} g_3^3 \\ & + d_{11} g_1^4 + d_{12} g_1 g_3^3 + d_{21} g_3 g_1^3 + d_{22} g_3^4 + e_{11} g_1^5 + e_{12} g_1 g_3^4 + e_{21} g_3 g_1^4 + e_{22} g_3^5 \end{aligned} \tag{S4}$$

$$\begin{aligned} y_3 = {} & \beta_0 + \beta_1 g_2 + \beta_2 g_3 + \beta_{11} g_2^2 + \beta_{12} g_2 g_3 + \beta_{22} g_3^2 + c_{11} g_2^3 + c_{12} g_2 g_3^2 + c_{21} g_3 g_2^2 + c_{22} g_3^3 \\ & + d_{11} g_2^4 + d_{12} g_2 g_3^3 + d_{21} g_3 g_2^3 + d_{22} g_3^4 + e_{11} g_2^5 + e_{12} g_2 g_3^4 + e_{21} g_3 g_2^4 + e_{22} g_3^5 \end{aligned} \tag{S5}$$

Next, the output gene (G) was calibrated based on the handling calibrated dataset (the hidden layer) (y_1 to y_3). So, the second calibrating process was utilized to predict the output gene expression (G) (**Fig. 1d and Fig. 2b**). Using these estimated nonlinear forms of the elements (**Supplementary Eq. S3** to **Supplementary Eq. S5**) and the nonlinear polynomial basis functions (**Supplementary Eq. S6**), we can, therefore, predict the expression of the output (target) gene (G).

$$\begin{aligned} G = {} & \beta_0 + \beta_1 y_1 + \beta_2 y_2 + \beta_3 y_3 + \beta_{11} y_1^2 + \beta_{12} y_1 y_2 + \beta_{13} y_1 y_3 + \beta_{22} y_2^2 + \beta_{23} y_2 y_3 + \beta_{33} y_3^2 \\ & + c_{11} y_1^3 + c_{12} y_1 y_2^2 + c_{13} y_1 y_3^2 + c_{21} y_2 y_1^2 + c_{22} y_2^3 + c_{23} y_2 y_3^2 + c_{31} y_3 y_1^2 + c_{32} y_3 y_2^2 + c_{33} y_2^3 \\ & + d_{11} y_1^4 + d_{12} y_1 y_2^3 + d_{13} y_1 y_3^3 + d_{21} y_2 y_1^3 + d_{22} y_2^4 + d_{23} y_2 y_3^3 + d_{31} y_3 y_1^3 + d_{32} y_3 y_2^3 + d_{33} y_2^4 \\ & + e_{11} y_1^5 + e_{12} y_1 y_2^4 + e_{13} y_1 y_3^4 + e_{21} y_2 y_1^4 + e_{22} y_2^5 + e_{23} y_2 y_3^4 + e_{31} y_3 y_1^4 + e_{32} y_3 y_2^4 + e_{33} y_2^5 \end{aligned} \tag{S6}$$

Using this relation (**Supplementary Eq. S6**), the output gene (G) was calibrated based on the nonlinear predicted input data (the hidden layer y_1 to y_3) (**Supplementary Eq. S3** to **Supplementary Eq. S5**). This nonlinear form of the mathematical formulation (**Supplementary Eq. S6**) provided a highly nonlinear relation compared to the linear format in **Supplementary Eq. S1** and nonlinear basis second-order form

in **Supplementary Eq. S2**. As seen in **Supplementary Eq. S3** to **Supplementary Eq. S5**, the five-order polynomial basis functions were applied in the first-calibration procedure to get the hidden layer elements. The calibrated input data (the hidden layer y_1 to y_3) provided a nonlinear relation between the output gene (G) and the input gene (g_1 to g_3). This type of modeling approach could separate the effect of each element (y) which was calibrated using the actual input data (g). But, the MLR and RSM could not offer such a conclusion in their calibrated procedures[15].

3.2 The procedure of the calibration.

The main effort of the gene calibration process (**Supplementary Eq. S1**, **Supplementary Eq. S2** and **Supplementary Eq. S3** to **Supplementary Eq. S6**) was to find the unknown coefficients that best fit the mathematical model on the database. We used the ordinary least squares (OLS) to determine the unknown coefficients of the polynomial functions in **Supplementary Eq. S1**, **Supplementary Eq. S2** and **Supplementary Eq. S3** to **Supplementary Eq. S6**. The errors between the predicted data of gene (G) and the estimated polynomial basis function ($f(\boldsymbol{x}) = p(\boldsymbol{x})\boldsymbol{\beta}$) was obtained using the following relation (**Supplementary Eq. S7**)[17]:

$$\varepsilon = [\boldsymbol{G} - p(\boldsymbol{x})\boldsymbol{\beta}]^T\,[\boldsymbol{G} - p(\boldsymbol{x})\boldsymbol{\beta})] \qquad \text{(S7)}$$

where T is the transpose operator. $\boldsymbol{\beta}$ is the vector of unknown coefficients. $p(\boldsymbol{x})$ is the polynomial basis functions obtained using i_{th} dataset in which $p(\boldsymbol{x})_{\boldsymbol{i}} = [1,\, g_1,\, g_2,\, g_3]_i$ for MLR by **Supplementary Eq. S1**, $p(\boldsymbol{x})_{\boldsymbol{i}} = [1,\, g_1,\, g_2,\, g_3,\, g_1^2,\, g_1 g_2,\, g_1 g_3,\, g_2^2,\, g_2 g_3,\, g_3^2]_i$ for RSM by **Supplementary** Eq. S2, and $p(\boldsymbol{x})_{\boldsymbol{i}} = [1,\, g_1,\, g_2, g_1^2, g_1 g_2, g_2^2, g_1^3, g_1 g_2^2, g_2 g_1^2, g_2^3, g_1^4, g_1 g_2^3, g_2 g_1^3, g_2^4, g_1^5, g_1 g_2^4, g_2 g_1^4, g_2^5]_i$ for the first element of the hidden layer y_1 by **Supplementary Eq. S3**, etc. The unknown coefficients can be directly approximated by minimizing the error and using the following relation (**Supplementary Eq. S8**)[18]:

$$\boldsymbol{\beta} = [p(\boldsymbol{x})^T p(\boldsymbol{x})]^{-1} [p(\boldsymbol{x})^T \boldsymbol{G}] \quad \text{(S8).}$$

Thus, the calibrated data can be obtained by approximating the unknown coefficients in **Supplementary Eq. S8** for the dataset $\boldsymbol{x}_t$ using the following relation (**Supplementary Eq. S9**):

$$G = p(\boldsymbol{x}_t)([p(\boldsymbol{x})^T p(\boldsymbol{x})]^{-1} [p(\boldsymbol{x})^T \boldsymbol{G}]) . \quad \text{(S9)}$$

The calibration process of the MLR, RSM, and MLRSM models was done using the same simple procedure; while, the high-order polynomial basis function was based on a two-step process in MLRSM and provided high-performance flexibility for the calibration of genes. Thus, the hidden layer-based dataset of genes can show the effect of a dataset on detection of the calibrated genes using a highly nonlinear relation. The best agreement of the input dataset of genes can be extracted by this type of modeling approach in the calibration process. So, the nonlinear correlations among the genes can be evaluated more accurately based on the nonlinear form of the polynomial basis function compared with the traditional RSM and MLR models.

3.3 Akaike Information Criteria (*AIC*)

AIC, as an alternative criterion, was utilized to select the effective input genes on the target gene. The null hypothesis of an input gene can be examined based on *P*-values and ΔAIC by Murtaugh's relation as follows[19]:

$$P = \Pr(\chi_k^2 > \Delta AIC + 2k) \Rightarrow \Delta AIC = F_{\chi_k^2}^{-1}(1-P) - 2(k) \quad \text{(S10)}$$

Where, *P* is the *P*-value for the null hypothesis of input genes which indicated removing all set of the hidden elements which are connected to the null input gene. *k* is the number of null genes and χ_k^2 is chi-square distribution function where *k* is degrees of freedom obtained 1.84 for *k*=1 and *P*=0.05[19]. ΔAIC is the differences in Akaike's information criterion based on all input genes ($RMSE_1$) with (*n*)-variable (i.e., 3) and null gene ($RMSE_2$) with (*n-1*)-variable (i.e., 3) where *RMSE* is root mean square errors as $\Delta AIC = 4Ln(RMSE_1) - 3Ln(RMSE_2)$[20], and *Ln* is the logarithmic operator. If ΔAIC is less than the chi-

square statistics (e.g. 1.84 for k=1 and P=0.05) then the input gene ith can be removed from the input data set for modeling target gene.

3.4 User-friendly codes employed for MLRSM scenario 2 in MATLAB.

We implemented our approach in MATLAB using approximately 120 lines of code as shown in the following lines.

```
clc
clear all
load data % data of genes for calibration
NG=1 % number of target gene
%% data in input and output basis genes
for i=1:size(X,2)
    if i~=gene
        x(:,k)=X(:,i);
    else
        y(:,1)=X(:,gene);
    end
end
nb=size(x,1);
% give the pattern of genes for the hidden elements
in=[1 2
    1 3
    1 4
    2 3
    2 4
    3 4];
%########################
Y=y(1:nb,1);
for j=1:size(in,1)
    for i=1:size(in,2)
        S(:,i)=xx(1:nb,in(j,i));
    end
clear model1
model1=RSM(S,Y);% calibrate in the first stage to give the hidden elements
aa(:,j)=model1;
X1(:,j)=PRSM(S,model1);% predicted handling data for target gene
end
model=RSM(X1,Y); %calibrate the target gene using hidden handling data
Yp=PRSM(X1,model); %predicted the target gene
%########################subroutine RSM###############

function [a]=RSM(x,y)
np=size(x,1);
nv=size(x,2);
for p=1:np
```

```
    m=1;
    for i=1:nv+1
        if m==1
            PX(p,m)=1;
        else
            PX(p,m)=x(p,i-1);
        end
        m=m+1;
    end
    if or>=2
        for i=1:nv
            for j=i:nv
                PX(p,m)=x(p,i)*x(p,j);
                m=m+1;
            end
        end
        if or>=3
           for i=1:nv
            for j=1:nv
                PX(p,m)=x(p,i)^2*x(p,j);
                m=m+1;
            end
           end
        if or>=4
           for i=1:nv
            for j=1:nv
                PX(p,m)=x(p,i)^3*x(p,j);
                m=m+1;
            end
        end
        end
        end
        end
    end
end
a=(PX'*PX)^-1*(PX'*y);
end
%########################subroutine PRMS ##########################

function Ev=PRSM(xv,a)
npv=size(xv,1);
nv=size(xv,2);
for p=1:npv
    m=1;
    for i=1:nv+1
        if m==1
            PXv(p,m)=1;
        else
            PXv(p,m)=xv(p,i-1);
        end
        m=m+1;
```

```
    end
    if or>=2
        for i=1:nv
            for j=i:nv
                PXv(p,m)=xv(p,i)*xv(p,j);
                m=m+1;
            end
        end
        if or>=3
           for i=1:nv
            for j=1:nv
                PXv(p,m)=xv(p,i)^2*xv(p,j);
                m=m+1;
            end
           end
        if or>=4
           for i=1:nv
            for j=1:nv
                PXv(p,m)=xv(p,i)^3*xv(p,j);
                m=m+1;
            end
        end
        end
        end
        end
        end
        end
Ev=PXv*a;
end
```

3.5 Supplementary Tables

Genes	X_{mean}	S_x	C_V	C_{sx}	X_{min}	X_{max}	X_{max}/X_{min} ratio
IL1B	2.41 E-3	4.93 E-3	2.056	2.089	4.52E-8	0.0196	4.34E+5
TNFA	7.42 E-3	1.09 E-2	1.468	2.626	4.26E-5	0.0547	1.28E+3
TLR4	5.93E-4	4.23E-4	0.713	0.997	1.66E-6	0.0021	1.27E+3
IL10	4.30E-5	8.57E-5	1.991	3.107	2.31E-10	4.92E-4	2.13E+6
IL4	4.11E-5	3.57E-5	0.870	1.698	1.012E-7	2.13E-4	2.10E+3

Supplementary Table S1. Statistical analysis of the mRNA expression datasets. X_{mean} is the mean (average); S_x is the standard deviation of variables; C_V is the coefficient of variations as X_{mean}/S_x; C_{sx} is the skewness of the data; X_{min} and X_{max} are respectively the maximum and the minimum of the data points.

Gene		Sequence of nucleotide (5'-3') *	Accession No.	Tm (C)	Product size (bp)
TLR4	F	CTTGCGTACAGGTTGTTCCTAA	NM_174198.6	56	153
	R	CTGGGAAGCTGGAGAAGTTATG			
IL1B	F	ATGAAGAGCTGCATCCAACA	NM_174093.1	56	196
	R	ATGGAAGACATGTGCGTAGG			
IL4	F	GCCACACGTGCTTGAACAAA	NM_173921.2	56	63
	R	TGCTTGCCAAGCTGTTGAGA			
IL10	F	TTCTGCCCTGCGAAAACAA	NM_174088.1	58	85
	R	TCTCTTGGAGCTCACTGAAGACTCT			
TNFA	F	TGACGGGCTTTACCTCATCT	NM_173966.3	56	221
	R	TGATGGCAGACAGGATGTTG			
ACTB	F	CCAAGGCCAACCGTGAGAAAAT	K00622	58	256
	R	CCACATTCCGTGAGGATCTTCA			

Supplementary Table 2. Bovine primers used in real-time PCR.
* F, forward; R, reverse

References

1. Das, R., Mitra, S. & Murthy C. A. Extracting gene-gene interactions through curve fitting. *IEEE Transactions on nanobioscience* **11,** 402–409 (2012).

2. Salleh, F. H., Zainudin, S. & Arif, S.M. Multiple linear regression for reconstruction of gene regulatory networks in solving cascade error problems. *Adv. Bioinformatics* 10.1155/2017/4827171 (2017).

3. Liu, Y., Luo, G. & Zhang, Y. Response surface modeling by local kernel partial least squares. IEEE Fifth International Symposium on Parallel Architectures, Algorithms and Programming. 10.1109/PAAP.2012.45 (2012).

4. Farahania, H. A., Rahiminezhad, A., Same, L. & Immannezhad, K. A comparison of partial least squares (PLS) and ordinary least squares (OLS) regressions in predicting of couples mental health based on their communicational patterns. *Procedia Social and Behavioral Sciences* **5,** 1459–1463 (2010).

5. Keshtegar, B. & Seghier, M. A. B. Modified response surface method basis harmony search to predict the burst pressure of corroded pipelines. *Eng. Fail Anal*. **89,** 177-199 (2018).

6. Maharjan, S. *et al*. Exploring codon optimization and response surface methodology to express biologically active transmembrane RANKL in E. coli. *PLoS ONE* **9,** e96259; 10.1371/journal.pone.0096259 (2014).

7. Sedlic, F. & Kovac, Z. Non-linear actions of physiological agents: Finite disarrangements elicit fitness benefits. *Redox Biol.* **13,** 235–243 (2017).

8. Cordell, H. J. Detecting gene-gene interactions that underlie human diseases. *Nat. Rev. Genet.* **10,** 392-404 (2009).

9. Félix, M. A. & Barkoulas, M. Pervasive robustness in biological systems. *Nat. Rev. Genet.* **16,** 483–496 (2015).

10. Solvang, H. K., Lingjærde, O. C., Frigessi, A., Børresen-Dale, A. L. & Kristensen, V. N. Linear and non-linear dependencies between copy number aberrations and mRNA expression reveal distinct molecular pathways in breast cancer. *BMC Bioinformatics*, **12,** 10.1186/1471-2105-12-197 (2011).

11. Valdetara, F. *et al*. A response surface methodology approach to investigate the effect of sulfur dioxide, pH, and ethanol on DbCD and DbVPR gene expression and on the volatile phenol production in Dekkera/Brettanomyces bruxellensis CBS2499. *Front. Microbiol.* **8,** 1727; 10.3389/fmicb.2017.01727 (2017).

12. Howard, R., Carriquiry, A. L. & Beavis, W. D. Application of response surface methods to determine conditions for optimal genomic prediction. *G3 (Bethesda)* **7,** 3103–3113 (2017).

13. Buettner, F. *et al*. Computational analysis of cell-to-cell heterogeneity in single-cell RNA-sequencing data reveals hidden subpopulations of cells. *Nat. Biotechnol.* **33,**155-160 (2015).

14. Meng, T. *et al*. Identification of differential gene expression profiles in placentas from preeclamptic pregnancies versus normal pregnancies by DNA microarrays. *OMICS* 16, 301-311 (2012).

15. Willmott, C. J. On the validation of models. *Phys. Geograph*. **2,** 184–194 (1981).

16. Wessa, P. Free statistics software, office for research, development and education, version 1.2.1., https://www.wessa.net (2017).

17. Lever, J., Krzywinski, M. & Altman, N. Points of significance: Principal component analysis. *Nat. Methods* **14,** 641–642 (2017).

18. Smith, L. I. *A tutorial on principal components analysis* (Cornell University Press, New York, 2002).

19. Lavine, M. Comment on murtaugh. *Ecology* **95**, 642-645 (2014).

20. Keshtegar, B. & Heddam, H. Modeling daily dissolved oxygen concentration using modified response surface method and artificial neural network: a comparative study**.** *Neural Comput. Applic.* 10.1007/s00521-017-2917-8 (2017).
